# Age-dependent genes in adipose stem and precursor cells affect regulation of fat cell differentiation and link aging to obesity via cellular and genetic interactions

**DOI:** 10.1186/s13073-024-01291-x

**Published:** 2024-01-31

**Authors:** Asha Kar, Marcus Alvarez, Kristina M. Garske, Huiling Huang, Seung Hyuk T. Lee, Milena Deal, Sankha Subhra Das, Amogha Koka, Zoeb Jamal, Karen L. Mohlke, Markku Laakso, Sini Heinonen, Kirsi H. Pietiläinen, Päivi Pajukanta

**Affiliations:** 1 Department of Human Genetics, David Geffen School of Medicine at UCLA, University of California, Los Angeles (UCLA), Gonda Center, Room 6357B, 695 Charles E. Young Drive South, Los Angeles, CA 90095-7088 USA; 2 Bioinformatics Interdepartmental Program, UCLA, Los Angeles, USA; 3 Department of Genetics, University of North Carolina at Chapel Hill, Chapel Hill, NC USA; 4 Department of Medicine, University of Eastern Finland and Kuopio University Hospital, Kuopio, Finland; 5 Obesity Research Unit, Research Program for Clinical and Molecular Metabolism, Faculty of Medicine, University of Helsinki, Helsinki, Finland; 6 HealthyWeightHub, Endocrinology, Abdominal Center, Helsinki University Central Hospital and University of Helsinki, Helsinki, Finland; 7 Institute for Precision Health, David Geffen School of Medicine at UCLA, Los Angeles, USA

**Keywords:** Aging, Obesity, Polygenic risk score (PRS), Adipose stem and precursor cells (ASPCs), Gene-age interactions

## Abstract

**Background:**

Age and obesity are dominant risk factors for several common cardiometabolic disorders, and both are known to impair adipose tissue function. However, the underlying cellular and genetic factors linking aging and obesity on adipose tissue function have remained elusive. Adipose stem and precursor cells (ASPCs) are an understudied, yet crucial adipose cell type due to their deterministic adipocyte differentiation potential, which impacts the capacity to store fat in a metabolically healthy manner.

**Methods:**

We integrated subcutaneous adipose tissue (SAT) bulk (*n*=435) and large single-nucleus RNA sequencing (*n*=105) data with the UK Biobank (UKB) (*n*=391,701) data to study age-obesity interactions originating from ASPCs by performing cell-type decomposition, differential expression testing, cell-cell communication analyses, and construction of polygenic risk scores for body mass index (BMI).

**Results:**

We found that the SAT ASPC proportions significantly decrease with age in an obesity-dependent way consistently in two independent cohorts, both showing that the age dependency of ASPC proportions is abolished by obesity. We further identified 76 genes (72 SAT ASPC marker genes and 4 transcription factors regulating ASPC marker genes) that are differentially expressed by age in SAT and functionally enriched for developmental processes and adipocyte differentiation (i.e., adipogenesis). The 76 age-perturbed ASPC genes include multiple negative regulators of adipogenesis, such as *RORA, SMAD3, TWIST2,* and *ZNF521*, form tight clusters of longitudinally co-expressed genes during human adipogenesis, and show age-based differences in cellular interactions between ASPCs and adipose cell types. Finally, our genetic data demonstrate that *cis*-regional variants of these genes interact with age as predictors of BMI in an obesity-dependent way in the large UKB, while no such gene-age interaction on BMI is observed with non-age-dependent ASPC marker genes, thus independently confirming our cellular ASPC results at the biobank level.

**Conclusions:**

Overall, we discover that obesity prematurely induces a decrease in ASPC proportions and identify 76 developmentally important ASPC genes that implicate altered negative regulation of fat cell differentiation as a mechanism for aging and directly link aging to obesity via significant cellular and genetic interactions.

**Supplementary Information:**

The online version contains supplementary material available at 10.1186/s13073-024-01291-x.

## Background

Aging is a biological process characterized by gradual deterioration of physiological systems. Over time, cells lose their abilities to proliferate, differentiate, and repair, leading to sustained wear and reduced organ performance [1]. Transformations also occur at the transcriptional level, with recent studies finding over 30% of genes to be differentially expressed (DE) with age in at least one of the following tissues: skin, subcutaneous fat, whole blood, lymphoblastoid cell lines [2]. For most prevalent diseases in the world, age is a dominant risk factor. Yet, despite consistent patterns of functional decline, the underlying biological and cellular mechanisms involved in aging vary across the body and are only partially understood [2, 3].

In adipose tissue, aging facilitates a preferential increase of the visceral fat depot relative to the subcutaneous adipose fat depot, and the rise of low-grade inflammation within the tissue [4, 5]. Furthermore, adipogenesis, the central reproductive process during which stem cells in the stromal vascular fraction differentiate into adipose stem and precursor cells (ASPCs) and subsequently form fat cells (i.e., adipocytes)[6], is known to decrease with age [7, 8]. While ASPCs themselves have been observed to undergo age-associated transformations, such as reduced lipid accumulation and a transition towards a macrophage-like state [9], complex factors underlying this decline in adipogenesis, including hormonal and micro-environmental alterations, and the involvements of specific ASPC genes remain poorly characterized in humans [5, 7–10].

Similar patterns of reproductive decline and tissue alterations have been observed in the adipose tissue of obese individuals, particularly in those with metabolically unhealthy obesity (MUO) [11, 12]. In MUO, the accumulation of excess fat is attributed to hypertrophy, or the presence of larger, inflamed adipocytes, which release proinflammatory cytokines into the adipose tissue and prematurely undergo cellular decline. By comparison, the efficient proliferation of smaller, generally well-functioning adipocytes (i.e. hyperplasia) drives metabolically healthy obesity (MHO) [13].

Although this decline of adipose tissue in MUO may partially be attributed to age, which is a well-known risk factor for obesity, the prevalence of obesity is increasing across all age groups [14, 15]. Previous studies have found general patterns of accelerated aging from obesity, but the effects of obesity on aging in not yet middle-aged adults, specifically in adipose tissue, remain largely unexplored [16, 17]. As ASPCs generate adipocytes, their numbers and differentiation capacities directly link to the metabolically healthy functions of adipose tissue. Thus, cellular composition-based analyses may provide new insight on the development of MUO. ASPC proportions have been observed to separately decrease with age and obesity, but it is not well understood how the two jointly affect the cellular percentages of ASPCs [18, 19].

Based on these previous studies [18, 19], we hypothesize that obesity already reduces the ASPC proportions in younger obese individuals, thus abolishing the known inverse correlation between age and ASPC numbers. To this end, we estimated ASPC proportions by integrating subcutaneous adipose tissue (SAT) bulk and single-nucleus RNA sequencing (snRNA-seq) data from several independent cohorts and tested these ASPC proportions for age effects while stratifying by the obesity status. We show that ASPC proportions differ by age among the normal weight individuals, while this age dependency is not seen among the obese individuals, who already at a young age have significantly lower ASPC proportions than the normal weight young individuals. Thus, our study demonstrates the aging effect of obesity on this key adipose cell type with adipocyte differentiation potential.

Adipogenesis is modulated by the expression of transcription factors driving the differentiation, such as PPARγ and the CEBPs [6]. Many adipocyte differentiating factors and their co-regulators exhibit altered expression in adipose tissue in response to age and obesity [20–22], suggesting that interactions between age and obesity may impact the transcriptomic profiles of ASPCs, due to the central role this cell type plays during the adipocyte differentiation (i.e., adipogenesis). To identify currently unknown ASPC genes involved in age-obesity interactions, we used adipose snRNA-seq data to first identify 72 ASPC marker genes and four TFs regulating ASPC marker genes that are DE by age in SAT ASPCs. We then investigated the longitudinal expression profiles and temporal co-expression of these 76 age-DE ASPC genes during human SAT ASPC differentiation, and how age influences ASPC ligand-receptor interactions between the 76 genes and genes in other adipose cell types. To assess the genetic risk contributions in these genes, we leveraged biobank data and built regional BMI polygenic risk scores (PRSs) in the UK Biobank (UKB), and discovered an obesity-dependent gene-age interactions from the variants residing within the *cis*-regions of the age-DE ASPC genes. Overall, our results identify the ASPC cell type as a driver of alterations in adipose tissue aging in response to obesity.

## Methods

### Finnish Twin Cohort (FTC) used for estimation of cell-type proportions and differential expression analyses

The Finnish Twin Cohort (FTC) consists of twins recruited through multiple longitudinal surveys beginning from 1975 by the Helsinki University Central Hospital, Helsinki, Finland [23–25]. The FTC study design was approved by the local ethics committee, and all participants gave written informed consent. In our study, we examined previously obtained subcutaneous adipose tissue (SAT) bulk and single-nucleus RNA-seq data from a total of 50 monozygotic (MZ) twin pairs (total number of individuals is *n*=100) who were BMI-discordant (BMI difference≥2.8 kg/m^2^) [26]. The mean age of these 50 twin pairs is 45.5 years (SD=17.7 years; 54% female), and the mean BMI is 29.1 kg/m^2^ (SD=5.8 kg/m^2^). The age of this group follows a bimodal distribution in that 56% of individuals are below 40 years old, while the remaining 44% are over 55 years old.

### METabolic Syndrome In Men (METSIM) cohort used for additional investigation of cell-type decomposition and differential expression analyses

The METabolic Syndrome In Men (METSIM) cohort consists of 10,197 Finnish males between the ages of 45 and 73, recruited through the University of Eastern Finland and Kuopio University Hospital, Kuopio, Finland [27]. The study design was approved by the local ethics committee, and all participants gave written informed consent. Detailed metabolic phenotypes were collected for all individuals, including cardiometabolic clinical measurements, fasting laboratory tests, and an oral glucose test [27].

To verify our findings from FTC [23–25], we examined bulk (*n*=335) and single-nucleus RNA-seq (*n*=84, a subset of the 335 individuals) data from SAT samples for a subset of randomly selected, unrelated METSIM men [27]. The mean age of the 335 men is 54.1 years (SD=4.9 years) and the mean BMI, 26.8 kg/m^2^ (SD=3.7 kg/m^2^). The mean age of the 84 men is 55.1 years (SD=4.9 years) and the mean BMI, 26.5 kg/m^2^ (SD=3.8 kg/m^2^).

### UK Biobank (UKB) used for GWAS and construction of BMI PRS

The UK Biobank (UKB) includes data collected since 2006 from 502,617 individuals aged 37 to 73 [28, 29]. Samples were collected across 22 different centers, and genotyping for over 800,000 variants was performed using one of either the Affymetrix or Applied Biosystems UK Biobank Axiom genotyping technology. Genotypes were then imputed with the Haplotype Reference Consortium and the merged UK10K and 1000 Genomes phase 3 reference panel [28, 29]. To account for potential confounding from relatedness and population structure, we restricted our analyses to the 391,701 individuals who were unrelated and of European ancestry. Data from UKB were accessed under application 33934.

### Processing of snRNA-seq data from FTC and identification of unique ASPC marker genes

SnRNA sequencing was previously performed on SAT biopsies of 6 unrelated individuals in FTC [23–25] using the 10X Chromium platform [30] and following the Single Cell 3′ v2 protocol [26]. These 6 individuals consist of three males and three females, with a mean age of 64.8 years (SD=4.6 years) and mean BMI of 26.2 kg/m^2^ (SD=3.4 kg/m^2^). Reads were aligned to the GRCh38 human genome assembly with GENCODE v38 gene annotations [31] and quantified using STARSolo in STAR [32, 33] v2.7.3a. Since snRNA-seq captures both pre-mRNA and exonic RNA, we used the command -soloFeatures GeneFull to generate counts for both exonic and intronic reads.

In each sample, we filtered out droplets with high extranuclear RNA using DIEM [34] filtering, which performs multinomial clustering and fixes low-count droplets as an empty cluster. The samples were then merged and processed with Seurat [35]. The merged data set was further filtered to only contain genes expressed in 3 or more cells and cells containing between 200 and 2500 detected genes and at most 5% mitochondrial expression. We then normalized, scaled, and corrected the counts for mitochondrial RNA reads using sctransform [36]. We identified 6 clusters in Seurat [35] using the top 15 principal components of the data and a resolution of 0.2.

Cell types were assigned to the clusters using SingleR [37]. We used SAT snRNA-seq data from 15 individuals [38], where clusters were manually annotated based on their marker genes, and the Database for Immune Cell Expression [39], which is included with SingleR [37], as reference datasets. We identified five broad cell types: adipocytes, ASPCs, myeloid cells, vascular cells, and T cells.

To identify unique ASPC marker genes, we first determined the marker genes for all five cell types. We used the FindAllMarkers function from Seurat [35] with default parameters and only.pos=TRUE to perform Wilcoxon rank sum tests and identify genes DE and upregulated for each cell type. Genes with a Bonferroni-adjusted *p*-value<0.05 were considered cell-type markers. We then removed ASPC marker genes that were also identified as markers for other cell types from the ASPC marker gene set.

### Alignment and gene quantification of bulk adipose tissue RNA expression data in FTC

Bulk RNA sequencing to read length of 75bp had previously been performed on SAT biopsies for 50 MZ, BMI-discordant twin pairs from FTC (*n*=100) [23–25] using the Illumina HiSeq2000 platform and Illumina Stranded mRNA preparation [26]. Read quality was first assessed using FastQC [40]. We aligned the paired-end bulk RNA-seq reads to the GRCh38 human genome assembly with GENCODE v38 annotations [31] using STAR [32] v2.7.8a with the two-pass method and default options. We then counted fragments at the gene level against the GRCh38 genome assembly with featureCounts [41] v2.0.2. Only uniquely mapped reads were retained. Technical metrics for the reads were obtained with the CollectRnaSeqMetrics command from Picard Tools v2.13.2 [42].

### Cell-type decomposition in bulk expression data from SAT

We estimated the cell-type proportions in processed SAT bulk expression data from the 50 twin pairs (*n*=100) from FTC [23–25] using the reference-based approach of Bisque with default parameters. Briefly, we used Bisque [43] to first train a reference expression profile from annotated SAT snRNA-seq data from 6 individuals out of the 50 pairs. The count matrices of bulk samples were then decomposed with the trained reference.

To assess cell-type proportion differences, we first grouped the individuals by age, BMI status, and sex. We based our age grouping on the bimodal age distribution of this subset, so that the individuals of age below 40 years were classified as younger, and the rest, who were all over 55 years, were classified as older. Because these individuals are BMI-discordant twins, we created our BMI status groups by classifying the lower BMI twin per pair as lower BMI and higher BMI twin as higher BMI. We performed two-sided Wilcoxon tests to compare the cell-type proportions between the 2 groups, using Wilcoxon *p*-value<0.05 as the threshold for a significant difference.

### SnRNA-seq of SAT biopsies from METSIM

For snRNA-seq, we processed SAT biopsies that were snap-frozen and stored in −80°C. A total of 84 samples [44] from the bulk SAT RNA-seq METSIM cohort (*n*=335) [27] were used for snRNA-seq. To increase sample size while reducing cost, we multiplexed 4 samples into a single channel and assigned nuclei to individuals using genotype data (see below). To perform snRNA-seq, we first isolated nuclei from frozen tissue. Adipose tissue was minced over dry ice and placed into lysis buffer (0.1% IGEPAL, 10 mM Tris-HCl, 10 mM NaCl, 3 mM MgCl_2_ in nuclease-free water). The tissue lysate was filtered through a 30-μm MACS SmartStrainer, further filtered with PBS, and centrifuged 500×*g* for 5 min at 4°C. The pellet was suspended in wash buffer (1.0% BSA in 1× PBS) and further filtered with a 40-μm FlowMi tip strainer. The nuclei were centrifuged, re-suspended in wash buffer, and immediately processed for library construction using the 10X Chromium v3.1 kit . Single-cell libraries were then sequenced on an Illumina NovaSeq at a target depth of 400 million reads per library.

### Alignment of METSIM snRNA-seq data

To align reads to the genome, we used STARSolo in STAR [32, 33] v2.7.9a, aligning against the GRCh38 human genome and GENCODE v26 gene annotations [31]. We included the CellRanger4 adapter trimming to remove polyA tails and TSO adapters. Gene expression was estimated from unique molecular identifier (UMI) counts against the full gene (including exons and introns).

To remove empty droplets and only keep those with nuclei, we ran DIEM [34] filtering. As cytoplasmic ambient RNA consists of a greater percentage of spliced RNA than nuclear RNA, we ran clustering on spliced, unspliced, and ambiguous UMI counts. Gene-UMI counts for spliced, unspliced, and ambiguous were combined into a single matrix as input. Then, we ran DIEM [34] with 10 clusters and added a prior count of 1 to the cluster and gene probabilities. Additional low-count clusters were assigned as empty clusters as well, and droplets belonging to the fixed and assigned empty clusters were removed.

As samples from four individuals were pooled together into one 10X channel for sequencing, we ran demuxlet [45] after sequencing to de-multiplex the nuclei to their individual sample IDs using the individuals’ genome-wide variant data prior to performing any analysis. Thus, the origin of each nucleus was traced back to one of the 4 individuals using the variants landing in the expressed regions detected in the snRNA-seq data and comparing those with the DNA-sample-based genotype data that we also had available for all of these individuals. The demuxlet tool [45] was run with default options, matching the UMI alleles against the hard-coded genotypes of the 4 samples in the corresponding run. Droplets with a singlet to doublet likelihood ratio greater than or equal to 2 were assigned as singlets.

### Clustering of METSIM snRNA-seq data

Before clustering, we further filtered and normalized the nuclei UMI count data. First, we removed droplets with less than 200 genes detected, over 5% of mitochondrial and over 1% of hemoglobin UMIs. Normalization for UMI depth was performed with sctransform [36]. As hemoglobin and mitochondrial transcripts represent extranuclear RNAs exogenous to the nuclei, we removed these genes from the list of variable genes used in clustering. To cluster the nuclei, we performed canonical correlation analysis (CCA) on the sctransform [36] normalized UMIs, as implemented in Seurat [35]. To reduce computational time, we selected 8 random samples as references for anchor pairing. For dimensionality reduction, we ran principal component analysis (PCA) on the corrected, integrated counts. Finally, to determine clusters, we ran the FindNeighbors and FindClusters Seurat [35] functions, using default parameters and the Louvain algorithm on 50 PCs. For visualization, we ran UMAP on the same set of PCs.

For cell-type identification, we first identified upregulated genes in each cluster using Seurat [35]. UMI counts were scaled to sum to 1000 per droplet and then log-transformed. Wilcoxon tests were run to assess statistical significance, and we considered significantly upregulated genes (Wilcoxon *p*<0.05) with a log_2_-fold change of at least 0.1 as cell-type markers. For cell-type identification, we manually curated assignments based on known markers.

### Additional investigation of ASPC proportion differences in METSIM

We used independent SAT expression data (*n*=335) from METSIM [27] to further investigate the differences observed in ASPC proportions in FTC. Reads of length 50bp were previously generated on the Illumina HiSeq2000 platform using the TruSeq unstranded library [46, 47]. We first aligned the reads to the hg19 genome assembly with STAR [32] v2.5.2 using the two-pass method and default options. Technical metrics were generated using the CollectRnaSeqMetrics command from Picard Tools v2.9.0 [42]. We filtered reads to remove those aligned to the mitochondrial genome, which have been shown to correlate with technical factors and expression of autosomal genes [26] and only retained uniquely mapped reads [38]. Gene counts were then calculated using featureCounts [41] v2.0.2.

We used the reference-based approach of Bisque [43] to perform cell-type decomposition on the 335 bulk samples, using the annotated snRNA-seq data from 84 of the 335 individuals [44] as the reference. As METSIM is an all-male cohort, we only performed comparisons by age and BMI status. We defined normal weight as BMI<25, overweight as 25≤BMI<30, and obese as BMI≥30. For our age groups, we used age below the 25th percentile (age≤51) and above the 75th percentile (age>58) as cutpoints for younger and older respectively.

### Correlating ASPC proportions with body composition

We assessed the relationship between the estimated ASPC proportions and fat mass and fat-free mass, as measured by impedance, in METSIM [27], using Spearman’s correlation and Spearman’s *p*<0.05 as the significance threshold. We tested the correlations four times: without any adjustments, adjusting for age, adjusting for BMI, and adjusting for both age and BMI. Proportions were centered and scaled to a mean of zero with unit variance, and outcomes were normalized by a rank-based inverse normal-transform.

### Motif enrichment analysis for SAT ASPC marker genes

We identified enriched transcriptional motifs in the promoter regions of each SAT ASPC marker gene using the findMotifs function of HOMER (Hypergeometric Optimization of Motif EnRichment) [48]. We specified 2 kb upstream and 1 kb downstream of the transcription start site (TSS) as the promoter region for each gene.

### Testing for DE by age in ASPCs

We tested the unique SAT ASPC marker genes and the TFs for which HOMER identified enriched motifs for DE by age in ASPCs, excluding the TFs identified as marker genes for other cell types. To increase the power of our testing, we used similarly processed and annotated SAT snRNA-seq data from 15 individuals [38, 49], 7 of whom had taken part in the FTC study [23–25] and 8 in the CRYO study [50, 51] for our testing. This 15-individual data set includes 10 females and 5 males, and the age ranges from 21 to 48 years with a mean age of 32.7 years (SD=7.1 years).

To test for DE by age, we compared gene expression levels between those below (*n*=8) and above (*n*=7) the median age of 34 years. We used the FindMarkers function in Seurat [35] with default arguments and logfc.threshold=0 to perform a Wilcoxon rank sum test for each ASPC marker gene and associated TF. Testing was limited to the droplets annotated as ASPCs, and the obtained *p*-values were adjusted for multiple testing with the Bonferroni method, using adjusted *p*<0.05 as the significance threshold.

We validated our DE results by similarly testing the significant genes for DE (*p*-adjusted<0.05) by age in the independent snRNA-seq data from SAT of 84 METSIM [27] individuals [44]. Leveraging the larger sample size of the METSIM cohort, we focused our DE analyses between the men whose ages fell in the lowest (age≤51 years, *n*=21) or uppermost quartiles (age>58 years, *n*=21). We performed three sets of tests: a BMI status-stratified testing, using BMI cut points for normal weight (BMI<25), overweight (25≤BMI<30), and obese (BMI≥30); an obesity-status-stratified testing, in which we considered BMI≥30 as obese and BMI<30 as non-obese; and a combined testing with all METSIM participants. *p*-values were adjusted for multiple testing using FDR<0.1.

### Protein-protein interaction and biological pathway analyses

We separately examined the 76 age-DE SAT ASPC genes and the remaining 79 non-age-DE SAT ASPC marker genes for protein-protein interactions using StringDB [52] and enrichment of associated biological processes using Webgestalt [53]. For the age-DE ASPC genes, we assessed relations between all 76 genes together, as well as separately examined the functional and physical relations between the 61 upregulated genes and 15 downregulated genes. We then subclustered the constructed protein interaction graph into individual networks using the mcl software [54] with an inflation parameter of 3.0, as in a previous study [55].

### Assessment of age-DE SAT ASPC genes in the visceral fat depot

To test whether the identified 76 age-DE SAT ASPC genes also show differences by age in ASPCs in the visceral fat tissue (VAT), we first downloaded publicly available single-nucleus RNA-seq data from 10 human visceral fat samples [56]. We performed DE testing on the 76 age-DE SAT ASPC genes between the individuals with age below (*n*=4) and above (*n*=6) the median age (41 years) similarly as in the SAT snRNA-seq datasets. We adjusted the *p*-values using FDR<0.05. We then tested the genes which showed consistent DE by age in both fat depots (adjusted *p*-value<0.05 and the same direction of expression change with age in both the SAT and VAT snRNA-seq cohorts) for enrichment of biological processes using Webgestalt [53].

### Correlating expression of the age-DE and non-age-DE SAT ASPC genes with metabolic phenotypes

We identified targeted outcomes for the 76 age-DE SAT ASPC genes and 79 non-age-DE ASPC marker genes by performing differential expression analyses for each set with the following key cardiometabolic phenotypes in METSIM [27]: BMI, waist-to-hip ratio (WHR), waist-to-hip ratio adjusted for BMI (WHRadjBMI), Homeostatic Model Assessment for Insulin Resistance (HOMAIR) Index, Matsuda index, fasting insulin, total triglycerides, total cholesterol, LDL cholesterol, interleukin-1 receptor antagonist (IL1RA), interleukin 1 beta (IL1B), C-reactive protein (CRP), alanine aminotransferase (ALT), systolic blood pressure, and fasting plasma glucose. We tested each outcome three times: without any adjustments, adjusting for only age, and adjusting only for BMI. To assess DE, we used bulk SAT RNA-seq data from 335 METSIM participants [46, 47]. To quantify transcript RNA abundance, we used Kallisto [57], which implements pseudoalignment to the transcriptome. The transcriptome index was created using GRCh38 GENCODE v26 transcript sequences [31]. Pseudoalignment was performed with default parameters. To obtain read and transcript per million (TPM) counts for genes, we summed the transcript isoform values of a gene for each sample. To perform DE testing across the metabolic traits, we used edgeR [58], including RIN, batch, and the first PC as covariates in the DE model. Kallisto [57] read counts per gene were rounded to integer values as required by negative binomial value of edgeR [58]. Finally, *p*-values were adjusted for multiple testing using FDR<0.05.

### Assay for transposase accessible chromatin (ATAC)-sequencing in human primary preadipocytes and data processing

We previously performed ATAC-sequencing on ASPCs isolated from the SAT biopsies of 9 twin pairs from FTC [23–25, 59]. As described earlier [59], we isolated the preadipocytes from the SVF fraction and followed the Omni-ATAC protocol. Libraries were sequenced on an Illumina HiSeq 4000 to produce an average of 45,021,302 (SD=8,419,051) reads. Sequencing reads were processed according to the ENCODE ATAC-seq Data Standards and Processing Pipeline. We first aligned reads to the hg19 genome using Bowtie2 [60] v2.2.9 with the following parameters: -k 4 -X 2000 –local. We then filtered out unpaired mapped reads and reads with MAPQ<30 as assessed by SAMtools [61], and only retained the uniquely mapped reads from the autosomes.

To call peaks, we used MACS2 [62] v2.2.7.1 and retained peaks with FDR<0.05. Peaks in blacklisted regions and those with fewer than one peak (bin) count per million mapped reads (BPM) in more than 10% of samples were filtered out. The log_2_-transformed peak TPMs were then corrected for family ID (as a random effect), age, sex, and fraction of reads in peaks (FriP), using the lme4 v1.1 R package [63].

### Differentiation of human primary SAT preadipocytes

Cryopreserved human primary white SAT preadipocytes (Zen-Bio catalog # SP-F-2, lot L120116E) were seeded into PromoCell Pad growth medium (PromoCell C-27410) with 1% Gibco Penicillin-Streptomycin (ThermoFisher 15140122) and cultured according to PromoCell Pad culturing protocols. Cells were maintained in a monolayer culture at 37°C and 5% CO_2_. Cells were propagated for the full experiment and not cultured beyond 5 passages.

The plating and differentiation of cells were staggered such that timepoints 1d, 2d, and 4d were collected at the same time, and timepoints 7d and 14d were collected at the same time. The 0d (ASPCs) timepoint was collected separately. To induce adipogenesis, cells were plated at confluency into 12-well plates for RNA-seq (4 isogenic biological replicates per time point) and the following day, adipogenesis was initiated using preadipocyte differentiation medium (PromoCell C-27436). The 1d and 2d timepoints were collected before any further media changes. For all other differentiation timepoints, 72 h after the preadipocyte differentiation medium was added, it was replaced with adipocyte nutrition medium (PromoCell C-27438), following PromoCell ASPC differentiation protocols.

### Bulk SAT RNA sequencing and processing of differentiating preadipocytes

For the RNA collection, cells were washed with PBS once and then lysed with TriZOL (Invitrogen 15596026), and RNA was purified using Direct-Zol RNA Mini-Prep (Zymo Research R2061). Libraries were prepared using the Illumina TruSeq Stranded mRNA kit and sequenced on one lane of an Illumina NovaSeq S1 flowcell to produce an average of 42 million (SD=5 million) reads per sample.

We aligned the reads to the GRCh38 genome with GENCODE [31] v39 annotations using STAR [32] v.2.7.10a. The two-pass method was used to account for novel splice junctions. We filtered the aligned reads to exclude reads mapped to the mitochondrial genome [26] or multiple transcripts [38]. We then used featureCounts [41] v2.0.3 to perform read summarization at the gene level. To verify the quality of the reads, we obtained quality control metrics for the RNA-seq data using FastQC [40] and Picard Tools v2.25 [42]. As an additional QC, we performed a PCA and observed the samples to form distinct clusters by timepoint (Additional file 1: Fig. S7).

### Testing the age-DE SAT ASPC genes for longitudinal DE and temporal co-expression during human adipogenesis

To assess the behavior during adipogenesis of the 76 age-DE SAT ASPC genes, we performed a longitudinal DE analysis using ImpulseDE2 [64] on the processed bulk expression data of the primary human preadipocytes in which we induced a 14-day differentiation [65]. We used the runImpulseDE2 function in case-only mode to first fits impulse models to the expression trajectories of each gene across the 14 days and then evaluate DE by performing log-likelihood ratio tests on each impulse model. *p*-values were adjusted for multiple testing using FDR<0.05.

To cluster the genes into subgroups based on their longitudinal expression patterns across the 6 timepoints, we used DPGP [66] with the following parameters: --cluster_uncertainty_estimate --check_convergence –check_burnin_convergence –true_times –alpha 2.0 -n 3000.

### Identification of ASPC ligand-receptor interactions

To determine ASPC ligand-receptor interactions, we first separated the SAT snRNA-seq data [38, 49] of the younger (*n*=8) and older (*n*=7) individuals using the annotated Seurat object into files containing the log-normalized counts and cell-type annotations per droplet per age group. On each file, we then ran the statistical analysis approach of CellphoneDB v4.0.0 [67] with default parameters to identify cell-cell interactions. After filtering the results to only include the interactions involving the age-DE SAT ASPC genes and ASPC cell type as at least one of the interacting cell types, we adjusted *p*-values for multiple testing using FDR<0.05. To compare the interaction scores, we used a paired, one-sided Wilcoxon test with Wilcoxon *p*<0.05 as the significance threshold, where we paired by interaction name, partners, ligand cell type, and receiver cell type and tested each partner cell type separately.

### Construction of PRS for BMI

Using the PRS guidelines outlined in [68], we developed polygenic risk scores (PRS) for BMI in males, females, and all unrelated Europeans from UKB [28, 29]. To ensure the normality of our trait in all groups, we inverse normal transformed each trait in two different ways: first in all individuals, and then for the males and the females separately.

We partitioned the cohort into 2 distinct groups: a base group for generating GWAS summary statistics, and a testing group for developing and applying the PRS model using penalized regression with summary statistics via lassosum [69] (see below). After evaluating the predictive power of various partitions with AVENGEME [70, 71], we split the cohort in half such that each group contained 50% to optimize the power in both PRS and PRS-age analyses. To prevent overfitting when applying the model, we used the split-validation approach, which further trains the polygenic risk score model on one half of the testing data before applying the new, optimized model to the other half. We partitioned the males and females separately so that the ratio of males to females was consistent across both groups.

### BMI GWAS in UKB

We obtained summary statistics for our traits of interest by performing genome-wide association studies (GWASs) in the male (*n*=90,045), female (*n*=105,818), and all individual (*n*=195,863) base groups. The analyses were conducted using the linear-mixed model approach implemented by BOLT-LMM v2.3.6 [72], where the top 20 genetic principal components, testing center, genotyping array, and for the overall group, sex, were included as covariates. For each base group, we performed two GWASs: one with only these covariates and another in which age and age^2^ were included as additional covariates. We excluded variants with MAF<1% and INFO<0.8 from the summary statistics.

### Building the BMI PRS model

We built the BMI PRS models using summary statistics from the BMI GWAS and genotype data from the testing groups (*n*_males_= 88,988, *n*_females_=104,614). The testing genotype data were filtered with plink v1.9 [73] to remove variants that were missing in 1% or more subjects, had MAF < 0.01, or violated Hardy Weinberg Equilibrium. We also removed individuals who were either missing more than 1% of genotypes, or exhibited extremely high or low heterozygosity, using the default values, as described earlier [68], to determine heterozygosity.

To construct the BMI PRS model, we used lassosum [69] to create a penalized linear regression model using the summary statistics and to optimize the model by training on the testing data. No *p*-value or linkage disequilibrium (LD) cutoffs were applied as they were not required for the penalized-based approach of lassosum [69]. Instead, to account for bias from LD, we supplied an external LD panel for hg19 and the European population [74]. Separate models were developed using all variants in the genome (i.e., the genome-wide BMI PRS), using just the variants in the *cis*-regions (within ±500 kb) of the 74 age-DE SAT ASPC genes located on autosomal chromosomes (i.e., the age-DE regional BMI PRS), and using just the variants in the *cis*-regions of the 78 non-age-DE SAT ASPC marker genes located on autosomal chromosomes (i.e., the non-age-DE regional BMI PRS).

We used the split-validation approach in lassosum [69] to apply the created models on the quality-controlled genotype data of the testing group and generate PRSs. Briefly, in the split-validation approach, the testing data are first split into two halves, and the existing PRS model is further optimized by training on one half before being applied to the other half. A second set of PRSs is then generated by switching on which half is being trained. The final scores are obtained by standardizing the two results.

### Assessment of the generated BMI PRSs for interaction with age

We used the generated genome-wide BMI PRS and the two regional BMI PRSs (built using the variants in the *cis*-regions of the age-DE SAT ASPC genes and of the non-age-DE ASPC marker genes) to test for PRS-age interactions on BMI in the unrelated Europeans from UKB [28, 29]. The BMI trait was first adjusted for the top 20 genetic PCs, testing center, genotyping array, and sex, and normalized through a rank-based inverse-normal transformation. We tested for a PRS-age interaction by creating the following model:

Adjusted BMI ~ Age + PRS + PRS × Age

We tested for interactions for all individuals using all three of the overall PRSs. For the genome-wide and age-DE regional BMI PRSs, we additionally examined the males and females separately using the respective sex-specific PRSs. To investigate obesity-related effects, we evaluated the interactions separately in the normal BMI (BMI<25) and obese (BMI≥30) individuals. To account for differences in the age distributions between the normal BMI and obese individuals, we performed frequency matching of the normal BMI and obese individuals by age year in males and females separately. This matching ensured consistency in the age distributions of the normal BMI and obese individuals while minimizing power loss. In each model, we performed Wald tests to assess the significance of the reported coefficients, with Wald-*p*<0.05 as the significance threshold.

To further evaluate the observed significance of the age-DE regional PRS interaction with age, we compared the age-DE regional PRS to the background genome by permuting the genome 10,000 times. In each permutation, we built regional BMI PRSs from 74 randomly selected autosomal genes and tested the created score for a significant PRS-Age interaction in BMI in the obese individuals of UKB (*n*=45,203). We evaluated significance by the ranking *p*-value observed in the original regional PRS (built using the variants in the *cis*-regions of the 74 autosomal age-DE ASPC genes) relative to the *p*-values observed in the 10,000 permutations, using ranking >95th percentile for significance.

### Identifying cis-regional variants interacting with age on BMI

In our single-variant interaction analysis, we assessed the variants in the *cis*-regions (±500 kb) of the age-DE ASPC genes for interactions with age on BMI by building the following model and performing a Wald-test on the Variant × Age coefficient for each variant separately:

Adjusted BMI ~ Variant + Age + Variant × Age

Here, the variant term corresponded to the number of copies of the allele positively associated with BMI in our non-age-adjusted BMI GWAS. To increase power, we limited testing to the variants which showed nominal significance in our non-age-adjusted BMI GWAS, passed LD-clumping with plink [73] v1.9, using an *r*^2^ cutoff of 0.1 and window of 250kb, and landed within ASPC open chromatin regions [59]. We furthermore examined only the same obese individuals who were included for the PRS-age interaction analysis. *p*-values were adjusted for multiple testing using Bonferroni <0.05.

## Results

### Obesity perturbs age-dependent changes in ASPC proportions

**Fig. 1:**
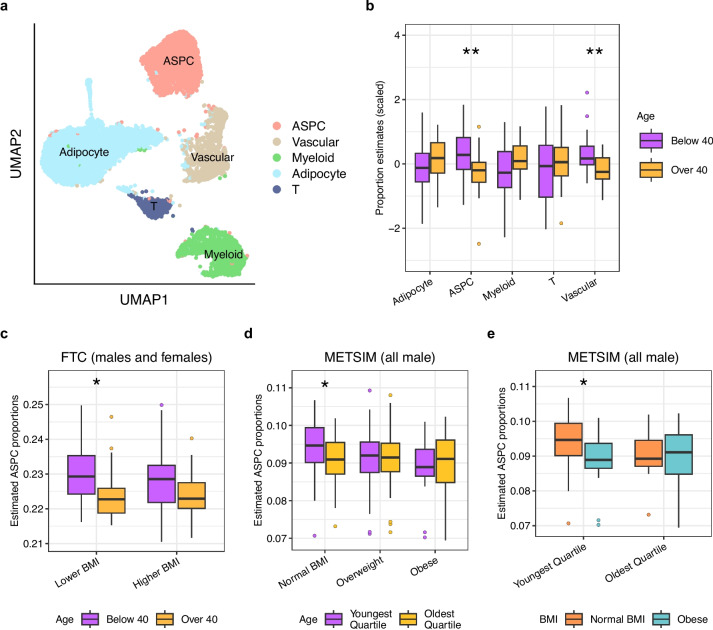
Comparisons of SAT cell-type proportion estimates by age and BMI status indicate that ASPC proportions decrease with age, and this difference is abolished by obesity. **a** Uniform Manifold Approximation and Projection (UMAP) visualization of 12,564 nuclei from SAT samples of 6 individuals from the Finnish Twin Cohort (FTC), colored by cell type. We assigned clusters to 5 major adipose cell types. **b** Boxplots comparing the centered and scaled SAT cell-type proportion estimates in bulk SAT RNA-seq from FTC between unrelated individuals below (*n*=28) and above 40 years of age (*n*=22) show a significant difference in ASPC proportions by age. The 40-year cutpoint was selected due to the bimodal distribution of age in FTC (see “Methods”). We randomly selected one individual per monozygotic (MZ) twin pair to ensure the individuals were unrelated. **c** Boxplots separate BMI-discordant MZ twin pairs from FTC into a lower BMI group consisting of the lower BMI twin per pair and higher BMI group with the higher BMI twin. Within each group, we compared ASPC proportions by age (*n*_lower BMI below 40_=28, *n*_higher BMI below 40_=28, *n*_lower BMI over 40_=22, *n*_higher BMI over 40_=22). **d** Boxplots separate normal BMI (BMI<25), overweight (25**≤**BMI<30), and obese (BMI**≥**30) individuals from the METSIM cohort and compare SAT ASPC proportions between those with age below the 25th percentile of age (*n*_normal BMI_=27, *n*_overweight_=46, *n*_obese_=19) and above the 75th percentile of age (*n*_normal BMI_=21, *n*_overweight_=34, *n*_obese_=18) in each group. **e** Boxplots separate the individuals from METSIM with age below the 25th percentile of age (*n*_normal BMI_=27, *n*_obese_=19) and above the 75th percentile of age (*n*_normal BMI_=21, *n*_obese_=18) and compare SAT ASPC proportions between normal BMI (BMI<25) and obese (BMI**≥**30) individuals in each age group. **b–e** Asterisks denote a significant difference in cell-type proportions between younger and older individuals as assessed by a Wilcoxon test. Significance thresholds for *p*-values: *p <0.05, **p <0.01, and ***p <0.001

Although age and obesity have been independently shown to reduce ASPC proportions, the mechanisms by which the two interact to impact ASPC abundance are largely unexplored. To investigate the connection between age, obesity, and cellular composition in adipose tissue, we estimated the proportions of the main cell types in bulk subcutaneous adipose tissue (SAT) expression data from 50 BMI-discordant MZ twin pairs in FTC [23–25] (see “Methods”). We created a reference expression profile by clustering SAT snRNA-seq data from 6 individuals of the 50 pairs [26] and assigning five main adipose cell types to the clusters (Fig. 1a). We then used reference-based Bisque [43] with the annotated SAT snRNA-seq data (see “Methods”) to obtain proportion estimates for these 5 main cell types in the 50 pairs (Additional file 2: Table S1). We assessed whether there are differences in the estimated cell-type proportions with age, using 40 years of age as the cut point between the younger and older group due to the bimodal age distribution in FTC (Fig. 1b; Additional file 2: Table S2), and randomly selecting one individual per twin pair to account for the twin status. We observed significantly higher (Wilcoxon *p*=3.8×10^−3^) ASPC proportions in the younger individuals (*n*=28) than in the older individuals (*n*=22) (Fig. 1b), which is consistent with previous studies [18].

We explored whether BMI influences these age-dependent changes in ASPC proportions. As the cohort consists of BMI-discordant MZ twins, we separated the twins into a lower BMI group consisting of the lower BMI twin per pair (*n*=50) and higher BMI group consisting of the higher BMI twin per pair (*n*=50). In each BMI status group, we compared ASPC proportions by age. In the lower BMI group, we found ASPC proportions to be significantly decreased with age (Wilcoxon *p*=6.4×10^−3^), but no such difference was observed in the higher BMI group (Fig. 1c; Table 1). To determine whether these age and obesity-based ASPC changes were sex-specific, we repeated our BMI status-stratified comparisons of ASPC proportions by age in the males and females separately. Both the lower BMI males (*n*=23) and lower BMI females (*n*=27) showed reduced ASPC proportions with age (Additional file 1: Fig. S1c, d); however, the stronger significance in females suggests that the joint effect of BMI and age on ASPC proportions may be more pronounced in the females.

**Table 1:** Comparisons of SAT ASPC proportions by age in the BMI status-stratified groups indicate significant differences by age in the lower/normal BMI individuals

**Cohort**^A^	**Group**^B^	***N***_**young**_^C^	***N***_**old**_^D^	**Mean ASPC proportion ± SD**^E^** of young**	**Mean ASPC proportion ± SD of old**	**W statistic**^F^	***p*-value**^G^
FTC	Lower BMI	28	22	0.230 ± 0.008	0.227 ± 0.011	409	0.0064
FTC	Higher BMI	28	22	0.225 ± 0.019	0.220 ± 0.014	373	0.1133
METSIM	Normal BMI	27	21	0.095 ± 0.007	0.090 ± 0.007	371	0.0431
METSIM	Overweight	46	34	0.091 ± 0.008	0.091 ± 0.008	808	0.8051
METSIM	Obese	19	18	0.091 ± 0.005	0.092 ± 0.006	96	0.3581

^A^Finnish Twin Cohort (FTC) or METabolic Syndrome In Men (METSIM) cohort

^B^The BMI status group used for comparisons of ASPC proportions by age

^C^Number of individuals in the younger group

^D^Number of individuals in the older group

^E^Mean ± standard deviation (SD) of ASPC proportions

^F^Wilcoxon test statistic between the ASPC proportions in the young and old individuals

^G^Wilcoxon test *p*-value between the ASPC proportions in the young and old individuals

### Additional investigation of the age and BMI-dependent differences in ASPC proportions

To elucidate the physiological implications of higher ASPC proportions and further examine the age and BMI-dependent ASPC proportion differences that we identified in FTC, we estimated the proportions of the SAT cell types (Additional file 2: Table S3) in bulk adipose expression data of 335 individuals [46, 47] from the all-male METSIM cohort [27] (see “Methods”) using reference-based Bisque [43] with snRNA data from 84 [44] of the 335 individuals. Leveraging the larger sample size and detailed phenotypes available in METSIM, we first correlated the ASPC proportions with fat and fat-free mass, which quantify the accumulation of fat mass related to adipose tissue and lean mass or non-adipose mass, respectively. We observed ASPC proportions to correlate negatively with fat mass (Spearman’s *ρ*=−0.16, *p*=3.3×10^−3^) and in turn positively with fat-free mass (Spearman’s *ρ*=0.16, *p*=3.1×10^−3^). These correlations remained significant after adjusting for age (*p*_fat mass_= 0.017, *p*_fat-free mass_=0.016), BMI (*p*_fat mass_= 2.1×10^−3^, *p*_fat-free mass_=2.1×10^−3^), or both BMI and age (*p*_fat mass_= 0.019, *p*_fat-free mass_=0.019). As reduced fat mass links to decreased risk against cardiovascular disease [75], these findings suggest a metabolic benefit to increased ASPC proportions.

Next, we partitioned the METSIM cohort by BMI to create a normal BMI, overweight, and obese group (see “Methods”), and in each of the three groups, we then tested for differences in ASPC proportions between the individuals in the lowest and highest age quartile. We found that the change in ASPC proportions with age in METSIM was affected by BMI in the same way as in the FTC cohort. In more detail, among the normal BMI men (*n*=48), the men in the youngest age quartile had higher ASPC proportions (Wilcoxon *p*=0.043) than the men in the oldest age quartile. We detected no significant differences in ASPC proportions in the overweight (*n*=80) or obese (*n*=37) METSIM men (Wilcoxon *p*>0.05) (Fig. 1d; Table 1). Down sampling the numbers of individuals to match sample sizes between the younger and older groups preserved the observed ASPC proportion differences in FTC and METSIM (Additional file 2: Table S4). The reduced significance of the difference in the normal BMI METSIM individuals when compared to FTC is also consistent with our sex-based analyses, given that METSIM consists only of males.

To determine whether there existed differences in ASPC proportions attributed to obesity and impacted by age, we compared the ASPC proportions between the normal BMI and obese individuals in the adipose bulk RNA-seq data of METSIM, testing separately in the individuals in the youngest and oldest age quartile. We observed that in the youngest age quartile group (*n*=46), the normal BMI men had significantly higher ASPC proportions than the obese men (Wilcoxon *p*=0.026) while no such significant difference was observed in the oldest age quartile group (*n*=39) (Wilcoxon *p*>0.05) (Fig. 1e).

Taken together, we see a metabolically likely beneficial higher ASPC abundance in young, normal BMI individuals that decreases with both age and obesity.

### Seventy-six SAT ASPC genes are DE by age

Since cell proliferation and differentiation are ultimately controlled by gene expression changes [6], and aging is known to alter gene expression in adipose tissue [3], we investigated transcriptomic changes in ASPCs resulting from age. We identified 151 unique SAT ASPC marker genes (Additional file 3: Table S5) in the SAT snRNA-seq data from 6 individuals of FTC [23–25] (Fig. 1a), and used HOMER [48] to find 21 transcriptional motifs enriched in the promoter regions of these SAT ASPC marker genes (Additional file 2: Table S6, S7). We evaluated these unique ASPC marker genes and the transcription factor (TF) genes observed at the enriched motifs for DE by age in ASPCs (Additional file 3: Table S5; Additional file 2: Table S6, S7). To increase the sample size of the ASPC DE analysis, we performed the DE testing in an independent snRNA-seq data set of 15 individuals [38, 49] from the FTC [23–25] and CRYO [50, 51] cohorts and compared gene expression between the younger and older individuals (see “Methods”). Of the 151 SAT ASPC marker genes and 21 TFs tested, 72 ASPC marker genes and 4 TFs (*SMAD3, STAT5A*, *STAT6*, *TWIST2*) regulating ASPC marker genes were significantly DE (adjP<0.05) between the two age groups, and most of these 76 age-DE ASPC genes (80.3%) significantly increased in expression with age (Additional file 3: Table S8).

### Verification of age-DE SAT ASPC genes in METSIM

To corroborate the findings from our DE testing, we assessed whether these 76 DE genes were also DE by age in ASPCs in SAT snRNA-seq data [44] between the individuals in the lowest (*n*=21) and highest (*n*=21) age quartiles from the METSIM [27] cohort. Considering the older age distribution in the METSIM cohort than in our discovery cohort (see the “Methods”) and the absence of females in the METSIM cohort, we found it encouraging that despite these differences, 31 (41%) of the 76 genes were also DE (FDR<0.1) in ASPCs from METSIM (Additional file 2: Table S9). When we then used the larger sample size of the METSIM cohort to evaluate whether BMI impacted the age-based DE in the SAT ASPC marker genes, we observed a similar effect as with age-based ASPC proportion differences seen in FTC and METSIM (Fig. 1c,d). Within the individuals without obesity (BMI<30), 37 (49%) of the 76 genes exhibited significant DE, i.e., passed FDR<0.1 in the same direction as in the discovery cohort (Additional file 2: Table S10). We furthermore found 26 genes to be significantly DE by age (FDR<0.1) with the same direction as in the 15-sample dataset in the normal BMI group (BMI<25), while no such DE genes were observed in either the overweight (25≤BMI<30) or obese (BMI≥30) BMI groups. Thus, consistent with the influence of BMI on age differences in ASPC proportions in FTC and METSIM, we see that obesity perturbs aging expression patterns in SAT ASPCs marker genes.

### Age-DE SAT ASPC genes and non-age-DE ASPC marker genes show distinct functional profiles

To functionally characterize the aging signatures in SAT ASPC gene expression, we next compared the 76 age-DE ASPC genes against the 79 non-age-DE ASPC marker genes. We observed significant enrichment for protein-protein interactions using the database STRING [52] among both gene sets (*p*_age-DE_=1.11×10^−16^, *p*_non-age-DE_<1×10^−16^), indicating the presence of biologically meaningful interactions. Within the age-DE genes, we found the significant PPI-enrichment to be driven by the 61 age-upregulated genes (*p*=8.88×10^−16^) since no significant enrichment was detected among the 15 age-DE genes downregulated by age (Additional file 1: Fig. S2a). We observed that the 61 genes showed significant enrichment (FDR<0.05) for the negative regulation of fat cell differentiation GO biological process (5 genes), and for age (10 genes), body weights and measures (33 genes, including 8 of the age-associated genes and 4 of the genes implicated in negative regulation of fat cell differentiation), and abnormal blood glucose homeostasis (7 genes) from the Human Phenotype Ontology database (Additional file 1: Fig. S2b). Within the observed protein network of these age-upregulated genes, we also identified 6 subclusters. Two of these six subclusters showed significant enrichment (FDR<0.05) for cell structure and elasticity, while two other subclusters were significantly enriched (FDR<0.05) for inflammatory responses and interleukin signaling (Additional file 1: Fig. S3). Thus, the 61 ASPC marker genes upregulated by age also seem to contain interacting networks of genes of molecular and clinical significance related to aging and obesity.

**Fig. 2:**
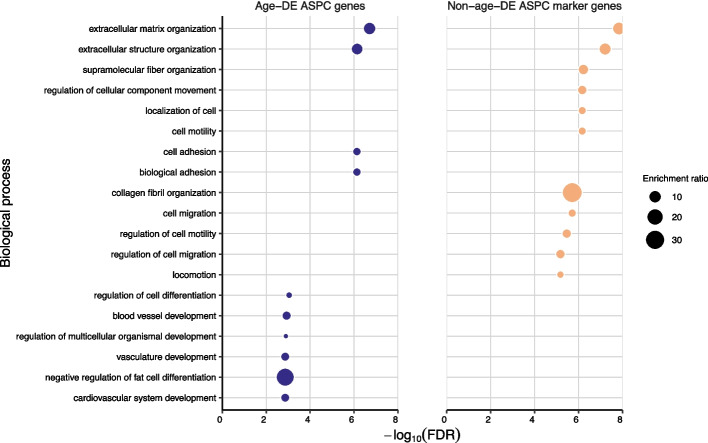
The 76 age-DE SAT ASPC genes show differences in functional pathways compared to the 79 non-age-DE ASPC marker genes. Dot plots compare the top 10 most significantly (FDR<0.05) enriched biological pathways for the 76 age-DE SAT ASPC genes (colored blue) and top 79 non-age-DE ASPC marker genes (colored orange). Each dot represents a significantly enriched pathway, where the size of dot represents the enrichment ratio. The remaining pathways of the 76 age-DE ASPC genes and 79 non-age-DE ASPC marker genes are shown in Additional file 3: Table S11 and S12, respectively

To identify the key functional pathways among the age-DE genes and how they differ from the non-age-DE genes, we searched for overrepresented biological processes in each gene sets using WebGestalt [53]. We found significant enrichments (FDR<0.05) for multiple organ and tissue developmental and differentiation-related functional pathways for the age-DE SAT ASPC genes, whereas for the non-age-DE ASPC marker genes, the most enriched pathways involved cellular structure and motility (Fig. 2). Moreover, the age-DE genes were uniquely and most highly enriched for negative regulation of fat cell differentiation (i.e. adipogenesis) (Fig. 2; Additional file 3: Table S11, S12). When we then assessed the up- and downregulated age-DE genes separately, we observed that the 61 age-DE genes, which increase their expression with age, drive these enrichments, including the highest enrichment for adipogenic inhibition. No significant enrichments were detected for the downregulated genes. Together, these differences in functional pathways indicate that the 76 age-DE genes include the more developmentally oriented subset of the SAT ASPC marker genes that also impacts regulation of adipogenesis.

**Fig. 3:**
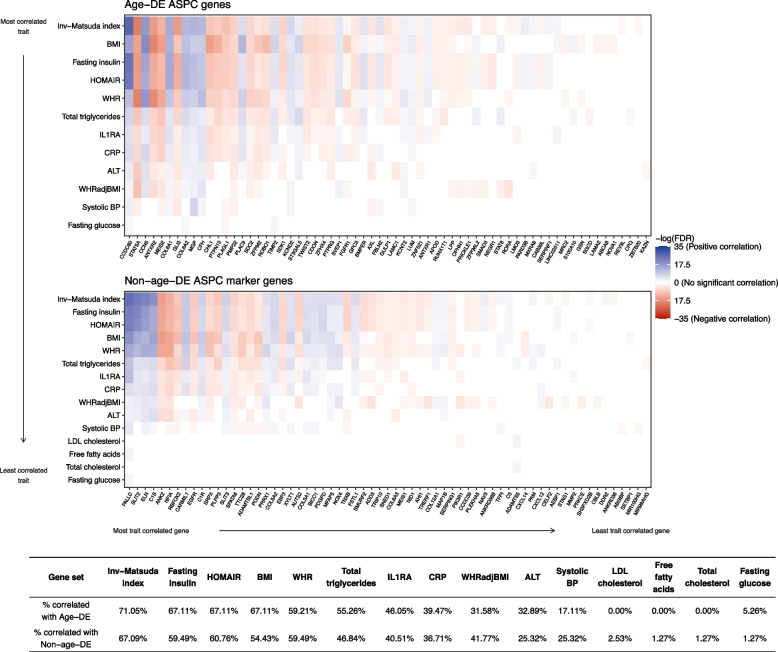
Age-DE SAT ASPC genes and non-age-DE ASPC marker genes show association patterns with many metabolic traits. Heatmaps compare the associations between the bulk expression of individuals genes in METSIM (*n*=335) and all tested metabolic phenotypes, for the 76 age-DE SAT ASPC genes (top) and 79 non-age-DE (bottom) ASPC marker genes. Traits and genes are both shown in decreasing order of number of significant correlations, as assessed by a Wilcoxon rank sum test. For each gene, we colored significantly associated traits (FDR<0.05) by directionality of the association, where a positive log_2_ fold change in gene expression represents a positive association. Red indicates a positive correlation, blue indicates a negative correlation, and genes colored black showed no significant correlations. Genes and outcomes which had no significant associations (FDR<0.05) were omitted. Below the heatmaps, we tabulate the proportions of genes per gene set that show significant associations with each metabolic trait

As adipose tissue function and plasticity are important for cardiometabolic health [11], we determined which cardiometabolic traits are correlated with the age-DE genes compared to the non-age-DE genes by using bulk SAT RNA-seq data from 335 individuals in METSIM [47] and performing DE analysis with 16 key obesity and related cardiometabolic phenotypes using FDR<0.05 for significance (see “Methods”). We observed generally similar association patterns within the two gene sets, with phenotypes related to insulin sensitivity and obesity (HOMAIR, Matsuda index, fasting insulin) ranking among the highest for the numbers of DE genes in both gene sets (Fig. 3; Additional file 3: Table S13, S14). Adjusting for age or BMI, which are known to correlate with many of the outcomes, resulted in smaller numbers of associations but still preserved the main association patterns in each gene set (Additional file 1: Fig. S4).

### A large, functionally relevant subset of the age-DE SAT ASPC genes are also DE by age in ASPCs from the visceral fat depot

As ASPCs are a key cell type in the visceral fat depot, which itself plays important roles in obesity and aging, we next investigated the 76 age-DE SAT ASPC genes for DE by age in the visceral adipose tissue (VAT). Using the publicly available single-nucleus RNA-seq data from 10 human VAT samples [56], we found that despite the smaller sample size of this VAT cohort (*n*=10), 36 (47.4%) of the 76 age-DE SAT ASPC genes show significant DE by age (FDR<0.05) in the same direction as in the 15 SAT samples (Additional file 3: Table S15). Next, we conducted a functional enrichment analysis with these 36 genes using Webgestalt [53] and observed that similarly as the 76 genes, these 36 genes also showed significant enrichment (FDR<0.05) for multiple developmental and differentiation-related pathways, and shared 8 significant pathways in common with the 76 age-DE SAT ASPC genes (Additional file 3: Table S11, S16).

To better understand the clinical significance of these age DE gene findings in both fat depots, we also searched for genetic support for involvement of these 76 age-DE SAT genes and 36 age-DE VAT genes in the diseases and traits using the Human Genetic Evidence (HuGE) scoring [76], available in the Common Metabolic Diseases Knowledge Portal. We found that 41 of the 76 (54%) and 22 of the 36 (61%) age-DE ASPC genes have HuGE scores with very strong or higher evidence (HuGE score≥30) for diabetes, lipid-related, or anthropomorphic outcomes, thus suggesting that lifelong genetic predisposition to obesity or metabolic disease may have already induced similar gene expression changes in ASPCs that typically occur during aging. Together, these results suggest that of the 76 age-DE SAT ASPC genes, a substantial subset (47.4%) is also DE by age in VAT, and that both age-DE ASPC gene sets are important for adipose tissue development and overall metabolic health.

### Longitudinal expression profiles of the 76 age-DE SAT ASPC genes identify seven temporally co-expressed gene subgroups during adipogenesis

**Fig. 4:**
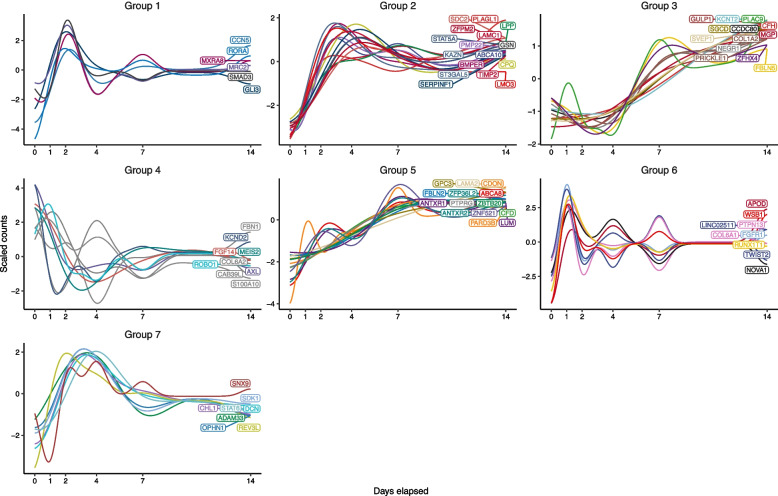
Longitudinal expression profiles of the age-DE SAT ASPC genes discover seven temporally co-expressed gene subgroups during adipogenesis. Plots of gene expression against time show the scaled predicted gene counts against the number of days elapsed since the start of the human SAT preadipocyte differentiation experiment. The predicted counts of each gene and gene groupings were obtained using DPGP, which fitted Gaussian models to each gene and performed clustering on the temporal expression data

We hypothesized that because the 76 age-DE genes are notably expressed in SAT ASPCs and enriched for regulation of fat cell differentiation, which is the key differentiation process in the fat tissue and declines and undergoes dysfunction with age [7, 8, 10, 21], these genes may link to changes in metabolic health via adipogenesis. Accordingly, we examined the behavior of the 76 genes during SAT ASPC differentiation. We used 4 isogenic biological replicates of human primary SAT ASPCs, in which we induced a 14-day adipocyte differentiation, extracted RNA at 6 adipogenesis timepoints and performed bulk RNA-seq with the 4 samples at the 6 adipogenesis timepoints over 14 days [65] (see “Methods”). Using ImpulseDE2 [64] to test the genes for DE across the 6 timepoints, we found 75 genes (99%) to be significantly DE during adipogenesis (FDR<0.05), supporting the notion that the expression of these genes significantly change during ASPC differentiation (Fig. 4; Additional file 3: Table S17).

We also observed that the impulse models showed similar longitudinal patterns between the expression profiles of several genes. This suggested that many of these genes may be co-expressed, commonly regulated, or functionally associated via shared pathways or TFs. We therefore used the DPGP tool [66] that clustered the genes into 7 subgroups based on their gene longitudinal co-expression patterns during the 14-day differentiation (Fig. 4; Additional file 3: Table S18).

Using EnrichR [77–79], we observed that 5 of the 7 subgroups (groups 1, 2, 3, 4, and 5) are significantly enriched (FDR<0.05) for the binding sites of adipogenesis and aging-linked TFs as reported in the human subset of the ChIP Enrichment Analysis (CHEA) 2022 database [80] (Additional file 2: Table S19): *ZNF217* [81] in groups 1 and 2; *SOX2* [82, 83] in groups 2 and 4; *TCF4* [84] in groups 3 and 5; *CTBP-1/2* [85] and *KDM2B* [86] in group 4; and TCF21 [87] in group 5. Noteworthy, subgroup 1 contains multiple previously established key negative regulators of fat cell differentiation: SMAD Family Member 3 (*SMAD3)*, Twist Family bHLH Transcription Factor 2 (*TWIST2*), and RAR Related Orphan Receptor A (*RORA*) [88–90]. Subgroup 1 also shows significant (FDR<0.05) enrichment for beta-catenin binding, a cellular mechanism repressing adipogenesis [91], using WebGestalt [53]. Thus, transcriptional regulation of adipogenesis and functional relatedness may drive the similarities in longitudinal gene expression patterns among the 76 age-DE genes during fat cell differentiation.

### The 76 age-DE SAT ASPC genes show differences in the strength of cell-cell interactions by age

As intercellular communication plays important roles throughout the process of adipogenesis [92], we next investigated differences by age in the ASPC ligand-receptor interactions involving the 76 age-DE SAT ASPC genes, using CellphoneDB [67] to identify and compare the significant ASPC-cell communications in the younger and older individuals of the SAT snRNA-seq cohort [49] (see “Methods”). We found significantly (Wilcoxon *p*<0.05) stronger cell-cell interactions in the adipose tissue of older individuals compared to the younger individuals, with the most significant differences being observed within ASPCs; between ASPCs and adipocytes; between ASPCs and T cells; and between ASPCs and perivascular cells, respectively (Additional file 2: Table S20). When we then ranked the cell-cell ligand-receptor interactions by the difference in strength in the older versus younger individuals to identify the key age-differing interactions, we observed that 7 of the top 10 interactions with greatest increase in interaction strength in the older individuals involved the age-DE SAT ASPC marker gene and adipogenesis repressor, *RORA* (Additional file 3: Table S21). These results suggest that age affects the cellular interactions of this well-known adipogenesis regulator TF, *RORA*.

### Age interacts with the regional polygenic risk score (PRS) of the age-DE genes in obese individuals

We next searched for a genetic relationship between local variants in the ASPC gene regions, age, and BMI in the unrelated Europeans from UKB [28, 29] and compared those to the results conducted similarly but with the genome-wide variants. We constructed the following PRSs for BMI in UKB: a genome-wide PRS generated using all variants in the genome, and regional PRSs generated using the variants in the *cis*-regions of the age-DE SAT ASPC genes and non-age DE ASPC marker genes (see “Methods”). Because we were interested in understanding aging-related mechanisms on BMI, the PRSs were built using GWASs without age adjustments (Additional file 1: Fig. S5a,b). In line with the previously created BMI PRSs [93], the genome-wide PRS explained 10.59% of variation in BMI whereas the age-DE and non-age-DE regional PRSs, which both included far fewer variants, explained 0.455 and 0.548%, respectively (Additional file 2: Table S22).

**Fig. 5:**
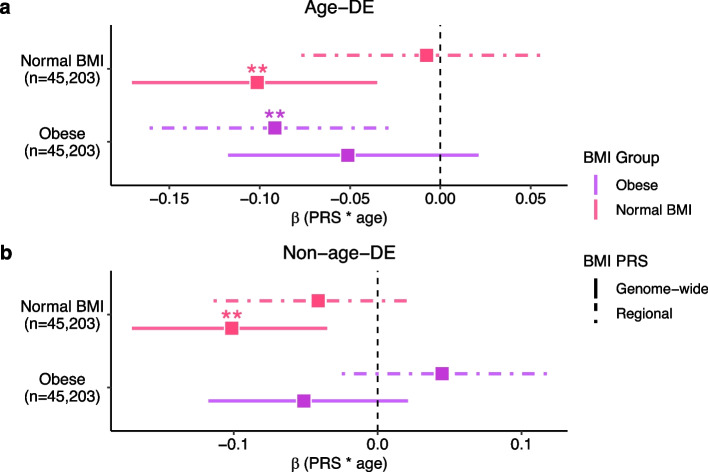
Age and the regional BMI polygenic risk score (PRS) comprising the local *cis* variants of the age-DE SAT ASPC genes interact negatively on BMI in obese individuals. Forest plots compare the 95% confidence intervals for the standardized estimated coefficient (**β**) of the age and BMI PRS interaction term in the linear model BMI ~ age + PRS + PRS × age between the genome-wide PRS, which includes all variants in the genome and a regional PRS, which includes the variants in the *cis*-regions of the (**a**) age-DE SAT ASPC genes and (**b**) non-age-DE ASPC marker genes. We separated the normal BMI (BMI<25) (*n*=45,203) and obese (BMI**≥**30) (*n*=45,203) individuals in UKB, and within each BMI group, evaluated the interaction term between age and the regional and genome-wide BMI PRSs. Each dot represents the mean estimate, and the horizontal bars denote the 95% confidence intervals of the estimate. Asterisks indicate that the age and PRS interaction term is significant in the model, as assessed by a Wald-test. Significance thresholds for *p*-values: *p <0.05, **p <0.01, and ***p <0.001

To search for genetic variants interacting with age, we created linear models consisting of age, PRS, and an interaction term between the age and PRS on BMI, examining separately the outcomes of normal BMI (*n*=45,203) and obesity (*n*=45,203). Intriguingly, we observed the age-DE regional PRS to have a significant negative interaction effect (*β*=−5.29×10^−3^, Wald-*p*=9.50×10^−3^) on BMI in the individuals with obesity, while no significant interaction with age on BMI was seen for the non-age-DE regional PRS (Fig. 5a, b; Additional file 2: Table S23). These results suggest the genetic risk from *cis*-regional variants of the age-DE ASPC genes on BMI is interacting with age in obese individuals. To formally confirm and further characterize this potential regional BMI PRS-age interaction in the obese individuals (Fig. 5a), we then performed a permutation analysis to evaluate whether such a significant PRS-age interaction on BMI was expected of regional PRSs built from randomly selected genes of the same size. Out of 10,000 regional PRSs, each constructed from the *cis-*regions of randomly selected gene sets of the same size as used to build the age-DE ASPC gene PRS, we observed only 5.3% to show significant PRS-age interactions (Wald-*p*<0.05) and only 0.98% to have a *p*-value below that of the age-DE ASPC gene PRS (Wald-*p*<9.50×10^−3^). These permutation results further show that in the obese individuals, age interacts with the regional BMI PRSs built using the *cis*-regional variants of the age-DE ASPC genes.

Given the heterogeneity of obesity [13], we next examined this identified age-DE regional PRS interaction for relation to metabolic disease. We first stratified the individuals with obesity in UKB into groups with MUO and MHO using two different MHO criteria, Meigs a criteria [94] and NCEP ATPIII criteria [95], and then separately evaluated the identified age-DE regional BMI PRS for an age interaction on BMI in each obesity group. Importantly, we found that under both definitions of MHO, only the individuals with MUO showed a significant PRS-age interaction (*p*_Meigs_=5.29×10^−3^, *p*_NCEP ATPIII_=6.84×10^−3^). We additionally stratified the obese individuals by type 2 diabetes (T2D) and observed that only the obese individuals with T2D showed a significant PRS-age interaction (*p*=8.29×10^−4^), in line with the MUO results. Taken together, these results suggest that the original regional PRS-age interaction on BMI that we observed in the obese individuals (Fig. 5a) is driven by individuals with MUO.

### Sex-specific analysis of the regional PRS-age interactions

Because we observed the ASPC proportion differences to be more significant in the females than in the males (Additional file 1: Fig. S1c, d), we also assessed whether the observed interactions between these genes, age, and obesity depended on sex by constructing the genome-wide and age-DE regional PRSs for the males (*n*=88,988) and females (*n*=104,614) separately with sex-specific summary statistics. We again separated the normal BMI and obese individuals in each sex and tested for the significance of the BMI PRS-age interaction term in a linear model for both the genome-wide and age-DE regional BMI PRSs. Similarly as in all obese individuals (Fig. 5a), the regional BMI PRS had a significant negative interaction in the obese females (Wald-*p*=7.68×10^−3^) (Additional file 1: Fig. S6; Additional file 2: Table S23), while no such significant interaction was observed in males (Wald-*p*>0.05). To investigate the effect of menopause on the observed regional PRS-age interaction, we tested the females in each BMI group by their reported menopause status, and observed that the regional PRS significantly negatively interacts with age in only the obese women already past menopause (Wald-*p*=0.038).

### Cis-regional variants of the age-DE SAT ASPC genes which reside within ASPC open chromatin contribute to interactions with age on BMI

To identify the individual variants which negatively interact with age on BMI, thus underlying the regional PRS interaction results, we performed interaction testing at the single-variant level using the same obese individuals (*n*=45,203) in UKB for whom we observed the significant regional BMI PRS-age interaction. To limit multiple testing, we focused our analyses on the independent, regulatory variants with the strongest main effects on BMI, thus testing only the LD-clumped *cis*-regional variants of the age-DE SAT ASPC genes that showed nominally significant (*p*<0.05) effects on BMI in the non-age-adjusted GWASs and landed within open chromatin regions of ASPCs[59]. The open chromatin regions were determined by ATAC-seq of human primary SAT ASPCs [59] (see “Methods”). Of the 124 variants tested, the variant *rs1755493*, which is located near the gene *CFD*, showed a significant negative interaction with age on BMI (Bonferroni-adjusted *p*<0.05). We found 3 additional SNPs with negative interactions passing nominal significance (*p*<0.05): *rs11631777*, located near the gene *RORA*; *rs113282909*, located near the gene *APOD*; and *rs17834271*, located near the gene *SERPINF1* (Additional file 2: Table S24). Taken together, our regional BMI PRS and single-variant analysis results provide genetic evidence connecting these 76 age-DE genes to gene-age interaction effects on BMI in individuals with obesity.

Overall, our integrative omics results identify the human ASPC cell type as a driver of alterations in adipose tissue aging in response to obesity and discover 76 such developmentally important SAT ASPC genes enriched for negative regulation of adipogenesis and impacted by both aging and obesity.

## Discussion

Age is a key risk factor for many cardiometabolic disorders, partly due to the critical metabolic function of adipose tissue that decreases over time [4–6, 8, 10]. Similar loss of function has also been observed in the adipose tissue of younger individuals with obesity [11–13]; however, it is not well understood how these responses to age and obesity relate to one another at the cell-type level in adipose tissue. In addition to being the precursors to fat cells, ASPCs undergo dramatic structural transformations in metabolically unhealthy obesity [4, 5, 9], making ASPC abundance and transcriptomic profiles noteworthy indicators of adipose tissue health. In this study, we used bulk and snRNA-seq data from SAT to show that young, normal weight individuals exhibit a high ASPC abundance which decreases with age. Of note, we observed that obese individuals do not exhibit this age-dependent abundance of ASPCs; rather, young, obese individuals already exhibit decreased SAT ASPC proportions when compared to young, normal weight individuals. We also observed a negative correlation between fat mass and ASPC proportions. As reduced fat mass has been connected to decreased risk of cardiovascular disease [75], our results suggest that the observed higher ASPC abundance we see in the young, lower BMI individuals may be metabolically beneficial. We then identify 76 such SAT ASPC genes DE by age that are enriched for multiple developmental processes and negative regulation of adipogenesis; alter gene expression levels during SAT ASPC differentiation; have stronger cell-cell interactions in the older than younger individuals; and contain regional DNA variants that interact with age on BMI in obese individuals in UKB. Taken together, this discovered set of 76 SAT ASPC genes implicates altered negative regulation of fat cell differentiation as a mechanism for aging and links obesity and aging via significant cellular and genetic interactions.

Despite being a main cell type in adipose tissue and directly linking to adipocyte abundance, human ASPCs are less investigated in bulk adipose decompositions, generally due to sparse reference data for the ASPC cell type [96]. Most previous studies on ASPC abundance rely on in vitro data [7, 8, 18, 19], which are underpowered to test for multi-variable effects. In our study, we integrated bulk SAT RNA-seq data with annotated snRNA-seq data from the same cohorts and with overlapping samples, to create robust adipose gene expression references, which we then used to accurately estimate ASPC proportions in the bulk SAT RNA-seq data. Leveraging the larger sample sizes and age distributions in the bulk expression datasets, we identified different aging patterns in adipose cellular composition based on the BMI status. The reduction of ASPC abundance with age has been detected in various mice and human cell line studies [18, 97]; however, here, we show the difference to be driven by the normal weight individuals by comparing them against the obese individuals. In a previous study examining ASPCs in adult women, the proportion of ASPCs in the stromal vascular fraction (SVF) of adipose tissue was found to be greater in the normal weight women compared to the obese women, although age dependencies were not explored. The study also noted that the adipocytes of the obese women were larger in volume and lower in numbers, thus implying an inverse relationship between ASPC abundance and fat mass [19], in line with our study that observed a direct inverse correlation between fat mass and ASPC proportions. Fat mass is also known to increase with age [98]. Thus, the findings of our study take the field forward by investigating the effects of age and obesity together on ASPCs and indicate that obesity prematurely induces the decrease in ASPC proportions that typically gradually occurs during aging in normal weight individuals.

We used our annotated adipose snRNA-seq data to characterize the aging profiles in ASPCs and show that aging induces substantial, transcriptomic changes in ASPCs, which may link to the age-related decline of adipogenesis [7, 8, 10]. We identified 72 ASPC marker genes, which comprise nearly one half of the ASPC marker gene population (47.7%), and 4 associated TFs, which are DE by age. These 76 genes are enriched for multiple organ and tissue development-related pathways and negative regulation of fat cell differentiation. Furthermore, particularly among the genes upregulated with age, which comprised the majority (80%) of the gene set, we found a high enrichment of inter-gene relations, which we decomposed into distinct subnetworks associated with collagen and inflammatory functions, respectfully. As the hallmarks of aging adipose tissue are increased fibrosis and inflammation promoted by immune dysfunction [4, 5, 9, 10], these detected enrichments suggest the upregulated genes as potentially important modulators underlying the aging induced changes in adipose tissue. Indeed, we also observed these age-upregulated genes to be linked to adipogenic inhibition, aging, and metabolic measurements, the latter two of which were dominated by those genes implicated for the negative regulation of fat cell differentiation. Among the upregulated age-DE genes is *ZNF521*, a master regulator of adipogenesis, whose repression is critical for adipogenesis, and the inhibition of which has been shown to trigger adipogenic commitment [99, 100]. The knockdown of *CCDC80*, the most downregulated gene during human adipogenesis in our study, has been previously shown to impact multiple key genes of adipose tissue function, including the major TF of adipogenesis, *SREBPF1* [101]*.*

By using 6-timepoint bulk RNA-seq data from a 14-day SAT ASPC differentiation experiment, we then extended relevance of adipogenesis to the set of 76 age-DE SAT ASPC genes, demonstrating the gene expression of nearly all (99%) of these genes to significantly change during the ASPC differentiation. We recognize that the changes observed during adipogenesis do not necessarily imply direct involvement in the differentiation process, but rather, only confirm the process as of biological interest for this gene set. This conclusion is also supported by the fact the non-age DE SAT ASPC marker genes were not enriched for negative regulation of fat cell differentiation. After performing clustering on the longitudinal expression data, we identified coordinated gene subgroups, which indicated temporal co-expression, common transcriptional regulation, or functional overlap linked to the longitudinal expression patterns of these genes during adipogenesis. In our identified subgroups, group 1 is of particular interest as it contains several genes previously reported to regulate adipogenesis (*RORA*, *TWIST2*, *SMAD3*, *CCN5*) [88–90, 102]. Additionally, this group showed significant enrichment for beta-catenin binding, a critical intercellular mechanism, through which Wnt-signaling acts to repress adipogenesis. Taken together, given the known decline of adipogenesis with age [7, 8, 10, 91], our findings provide direct candidate regulators of age-dependent adipogenesis in humans.

While the 76 age-DE genes were discovered in data originating from the subcutaneous fat depot, our study also links 36 of them to the aging ASPC profiles in the visceral fat depot. The visceral fat depot has been found as highly relevant for both aging and obesity, growing disproportionately compared to the subcutaneous fat depot in response to either [4, 5]. Overall, we found nearly half of these age-DE SAT ASPC genes to also undergo similar expression changes with age in VAT and showed that these genes DE in both fat depots retain the developmental and differentiation-related functional enrichments observed in the original age-DE SAT ASPC gene set. These shared functional enrichments, coupled with the high presence of metabolic disease-associated genes, suggest the presence of some coordination in the aging response relevant for metabolic health across the two main human fat depots.

In bulk SAT RNA-seq data from METSIM, we observed not only consistent differences in the ASPC proportions with age and obesity, but also significant associations between the expression of SAT ASPC genes and the key obesity and related cardiometabolic phenotypes. The most associated phenotypes were obesity and insulin sensitivity traits, in line with the established contributions of ASPC transcriptomic profiles to the dysmetabolic adipose tissue states observed in obese and type 2 diabetic individuals [59, 103]. Moreover, although we lacked sufficient power to examine obesity effects on the aging patterns of the ASPC genes in our discovery cohort, we were able to show a BMI-dependency in the DE consistency of the 76 age-DE SAT ASPC genes in METSIM. Consistent with the observed proportion differences, we found obesity to weaken the age-based gene expression changes in ASPCs. Thus, our findings support the identified ASPC genes as likely effectors of interactions between age and obesogenic cardiometabolic disorders.

Since the effects from interactions between genetic and environmental factors are typically relatively small [104], and further limited by the burdens of multiple testing, even cohorts as large as the UKB may lack adequate power for the detection of GxE effects with individual variants. In our study, using a regional PRS to represent the genetic signals across these age-DE SAT ASPC genes, we were able to identify a novel obesity-specific negative interaction between age and the regional BMI PRS. We found that the *cis*-regions of the age-DE genes are involved in unique gene-age interactions on BMI in obese individuals, thus showing that the presence of obesity may influence how these genes change their signals with age. Noteworthy, our interaction result was further confirmed by our permutation analysis of similarly sized random gene sets from the genome, and no interaction was observed with the regional PRS of the non-age-DE ASPC marker genes. Our single-variant analysis detected only one statistically significant variant with the same directionality of the interaction as seen in the regional PRS interaction analysis. This proposes that regional PRSs may provide a more powerful method for the discovery of novel multi-locus GxEs, which are often indiscernible at the single-variant level due to small effect sizes and multiple testing. Moreover, we discovered the negative gene-age interactions to stem from the obese individuals with MUO and T2D and not be observed in those obese individuals without MUO or T2D. These findings support not only the growing evidence of strong heterogeneity underlying obesity [13], but are also in line with our observed cardiometabolic trait associations in METSIM and enrichments for metabolic disease-associated genes.

Through our assessments of ligand-receptor interactions between ASPCs and other SAT cell types, we discover the TF *RORA* to result in the largest intercellular association differences, specifically greater in the older group. *RORA* is known to negatively regulate adipogenesis by targeting the key TFs driving the differentiation [88, 89], but to the best of our knowledge, its responses to age have not been previously well studied in the ASPC cell type. Here, our ligand-receptor assessments revealed an age dependency in cholesterol-based intercellular communications involving *RORA* between ASPCs and multiple SAT cell types, strengthened in the older individuals. As *RORA* plays inhibitory roles in cholesterol metabolism through a transcriptional network overlapping with its adipogenic inhibition [105, 106], its upregulated and increased intercellular interactions with age suggest a possible mechanism behind age-based dysfunction in adipose tissue. We also found that *RORA* is upregulated with age in ASPCs and that during SAT ASPC differentiation, the longitudinal expression of *RORA* temporally clusters with multiple other adipogenesis regulators, which together show significant enrichment for the inhibitory mechanism of adipogenesis via beta-catenin binding [91]. Furthermore, variant *rs11631777*, residing within an ASPC ATAC-seq peak in the cis-region of *RORA*, showed a negative interaction with age on BMI. Together, our converging results propose RORA as an important intercellular effector in adipose tissue of aging and its interactions with obesity.

Although our study provides insight on the impact of obesity on adipose tissue aging, there are several limitations to consider. The lack of completely controlled conditions, typical for any human observational study, may make our findings susceptible to other unaccounted environmental factors. Our analyses were also conducted using the imputed genome-wide SNP data in the European unrelated subset of UKB and the Finns in FTC, CRYO, and METSIM, meaning that our findings cannot be generalized to other ethnicities and that no rare variants were included in our study. In addition, neither METSIM nor UKB span full age ranges. Thus, cohorts with even larger age and BMI ranges are needed to more precisely study aging and obesity-driven behaviors in adipose tissue. Lastly, although evaluating the expression of the 76 genes in differentiating human primary ASPCs enabled us to identify their expression patterns associated with adipogenesis, we recognize the limitation that we were unable to directly profile ASPCs from the individuals used in the rest of our study and instead utilized commercially available human primary SAT ASPCs. In the future, it would be important to conduct differentiation experiments in ASPCs isolated from old and young adults to further understand the age-dependent role that each of these genes plays in ASPC differentiation.

## Conclusions

In conclusion, using snRNA-seq to estimate the main adipose cell-type proportions in bulk RNA-seq, we discover a metabolically favorable abundance in normal weight/control individuals’ ASPC proportions which decreases with age and is obstructed by obesity. We identify 76 developmentally important age-DE SAT ASPC genes, which we show to be linked to negative regulation of adipogenesis and age-dependent cellular interactions, centering around the TF *RORA*. We also demonstrate that the BMI effects of the *cis* variants residing around these differentially expressed ASPC genes are impacted by interactions with age in UKB. Together, our results identify the ASPC cell type as a driver of alterations in adipose tissue aging in response to obesity.

### Supplementary Information

**Additional file 1:** **Fig. S1.** Estimated SAT cell-type proportions in the Finnish Twin Cohort (FTC) differ by age, BMI status, and sex. (a) Boxplots compare the scaled cell-type proportion estimates in bulk SAT RNA-seq data from FTC by the BMI status (nlower BMI=50, nhigher BMI=50). The lower BMI twin per pair was classified as lower BMI for the pair and the higher BMI twin as higher BMI for the pair. (b) Boxplots compare the scaled cell-type proportion estimates from FTC between males (*n*=46) and females (*n*=54). (c,d) Boxplots compare the SAT ASPC proportions by age within the BMI status groups in (c) females (nbelow 40=34, nover 40=20) and (d) males (nbelow 40=22, nover 40=24) from FTC. (a-d) Asterisks denote a significant difference in cell-type proportions between the colored groups as assessed by a Wilcoxon test. Significance thresholds for *p*-values:*p <0.05, **p <0.01, and ***p<0.001. **Fig. S2.** The 76 age-DE SAT ASPC genes contain protein interaction networks with significant enrichments for age, metabolic outcomes, and adipose tissue development. (a,b) The inter-gene relations between (a) the 76 age-DE SAT ASPC genes and (b) 61 age-upregulated SAT ASPC genes are visualized as a knowledge graph. (a, b) Each node indicates a gene, and each edge indicates a relation connecting a set of genes, where edges are colored by relation type as follows: from a curated database (cyan), experimentally determined (magenta), gene cooccurrence (blue), co-expression (black), protein homology (lavender). (b) We shade the gene nodes in networks significantly enriched for key pathways and phenotypes of interest as follows:  body weights and measures (blue), age (red), lipid or lipoprotein measurement (green), abnormal glucose homeostasis (yellow), regulation of fat cell differentiation (magenta). The enrichment results are reported in the panel. **Fig. S3.** Clustering of the protein interaction network induced by the upregulated age-DE SAT ASPC genes identifies six subnetworks of inter-gene relations. (a-f) The clusters derived from the protein network of the 61 age-upregulated SAT ASPC genes are visualized as individual knowledge graphs. Each node indicates a gene, and each edge indicates a relation connecting a set of genes, where edges are colored by relation type as follows: from a curated database (cyan), experimentally determined (magenta), gene co-occurrence (blue), co-expression (black), protein homology (lavender). **Fig. S4.** Age and BMI influence association patterns between metabolic phenotypes and the bulk expression of both the age-DE ASPC genes and non-age-DE ASPC marker genes in the bulk SAT RNA-seq data from the METSIM cohort (*n*=335). Heatmaps display significant associations (FDR<0.05) between metabolic outcomes and the bulk expression of (a, b) the 76 age-DE ASPC genes and (c, d) the 79 non-age-DE ASPC marker genes. Each outcome has been adjusted for (a, c) age or (b, d) BMI. (a-d) Gene-outcome pairs are color-coded based on directionality and significance: red indicates a positive correlation, blue indicates a negative correlation, and white represents no significant correlation. Significance was assessed by a Wilcoxon rank sum test and a positive log2 fold change in gene expression with the outcome was defined as a positive association. We excluded genes and outcomes not identified in any significant gene-outcome pair. Below the panels, a table summarizes the proportions of genes per gene set that show significant associations with each adjusted outcome. Abbreviations are as follows: BMI indicates body mass index; HOMAIR Homeostatic Model Assessment for Insulin Resistance; C-reactive protein; IL1RA interleukin-1 receptor antagonist; ALT alanine transaminase; WHR waist-to-hip ratio; WHRadjBMI waist-to-hip ratio adjusted for BMI; and IL1b Interleukin-1 beta. **Fig. S5.** Age adjustments minimally alter the results of BMI GWASs and predictive performance of BMI PRSs in the UK Biobank. (a,b) A Miamiplot visualizes the effect of age adjustment on the summary level results of BMI GWAS using unrelated European individuals from UKB. Variants in the *cis*-regions of the age-DE ASPC genes are plotted by chromosomal position against the -log10(*p*-value) from BMI GWASs conducted with (a) only males (*n*=90,045) and (c) only females (*n*=105,818). The bottom panel reports the BMI GWAS where age and age2 were included as covariates, while in the BMI GWAS for the top panel, no age-related term was used as a covariate. A horizontal line marks the genome-wide significance threshold (*p*=5x10-8). (c) Paired bar plots compare the proportion of obese individuals (BMI>=30) in each PRS decile between the age-adjusted and non-age adjusted genome-wide BMI PRSs created using unrelated European individuals from the UKB (*n*=193,602). The error bars represent the 95% confidence intervals of the proportions. **Fig. S6.** Sex-stratified PRSs identify sex-specific effects on BMI and on age-obesity interactions in the *cis*-regions of the age-DE ASPC genes in the UK Biobank. (a,b) Forest plots compare the 95% confidence intervals for the standardized estimated coefficient (β) of the age and BMI PRS interaction term using the regional and genome-wide PRSs constructed for the (a) males (*n*=88,988) and (b) females (*n*=104,614) in UKB. We separated the normal BMI (BMI<25) and obese (BMI>=30) individuals in each sex. Asterisks indicate that the age and BMI PRS interaction term is significant in the model, as assessed by a Wald-test. Significance thresholds for *p*-values: *p <0.05, **p <0.01, and ***p <0.001. **Fig. S7.** PCA analysis of SAT adipogenesis time point data shows clustering by the timepoint. Biplot compares the first and second principal component (PC) of the expression data of each sample from the SAT ASPC differentiation experiment (nsamples=24, i.e. one sample in 4 isogenic biological replicates in six time points). We colored points by the time elapsed between the start of the experiment and when the sample was taken. PCs were calculated using the log2- transformed transcripts per million.

**Additional file 2:** **Table S1.** Summary of the estimated proportions of the 5 major subcutaneous adipose tissue cell-types in FTC (*n*=100). **Table S2.** Wilcoxon tests comparing proportions of the 5 major subcutaneous adipose tissue cell-types between the unrelated individuals below (*n*=28) and above 40 years old (*n*=22) in FTC. **Table S3.** Summary of the estimated proportions of the 20 major subcutaneous adipose tissue subcell-types/cell-types in METSIM (*n*=335). **Table S4.** Down sampling the numbers of individuals to match sample sizes between the younger and older groups preserves the observed SAT ASPC proportion differences in FTC and METSIM (see Table 1). **Table S5.** ASPC marker genes from SAT snRNA-seq data of 6 individuals in FTC (see the excel file Additional file 3: Table S5.xlsx). **Table S6.** Known motifs from HOMER enriched in promoter regions of the 151 unique SAT ASPC marker genes. **Table S7.** Motifs determined by *de novo *motif discovery in HOMER to be significantly enriched (*p*<1x10-10) in the promoter regions of the 151 unique SAT ASPC marker genes. **Table S8.** Seventy-six SAT ASPC genes (i.e. 72 ASPC marker genes and four transcription factor genes regulating ASPC marker genes) are significantly differentially expressed (DE) by age (FDR<0.05) using the ASPC data from the SAT snRNA-seq cohort (*n*=15) (see the excel file Additional file 3: Table S8.xlsx). **Table S9.** Our additional analysis in the independent METSIM cohort shows that 41% of the 76 age-DE SAT ASPC genes exhibit consistent DE (FDR<0.1 and the same direction as in the discovery snRNA-seq cohort) in the ASPCs from the subcutaneous adipose snRNA-seq data of the youngest and oldest age quartiles of the METSIM cohort. **Table S10.** Our additional analysis in the independent METSIM cohort shows that 49% of the 76 age-DE SAT ASPC genes exhibit consistent DE (FDR<0.1 and the same direction of effect as in the discovery cohort) in the ASPCs from the subcutaneous adipose snRNA-seq data of nonobese (BMI<30) individuals from the METSIM cohort (*n*=38).**Table S11.** The pathway tool WebGestalt identifies significantly (FDR<0.05) overrepresented developmental and differentiation-related Gene Ontology biological processes within the 76 age-DE SAT ASPC genes (see the excel file Additional file 3: Table S11.xlsx). **Table S12.** The pathway tool WebGestalt identifies significantly (FDR<0.05) overrepresented Gene Ontology biological processes within the 79 non-age-DE SAT ASPC genes (see the excel file Additional file 3: Table S12.xlsx). **Table S13.** Significant correlations (FDR<0.05) between the expression of the 76 age-DE SAT ASPC genes and cardiometabolic phenotypes using subcutaneous adipose bulk RNA-seq data from METSIM (*n*=335) (see the excel file Additional file 3: Table S13.xlsx). **Table S14.** Significant correlations (FDR<0.05) between the expression of the 79 non-age-DE SAT ASPC marker genes and cardiometabolic phenotypes using subcutaneous adipose bulk RNA-seq data from METSIM (*n*=335) (see the excel file Additional file 3: Table S14.xlsx). **Table S15.** Nearly half (47.4%) of the 76 age-DE SAT ASPC genes show significant DE by age (FDR<0.05 in the same direction as in the SAT snRNA-seq cohort) in VAT ASPCs from a publicly available VAT snRNA-seq data of 10 individuals (see the excel file Additional file 3: Table S15.xlsx). **Table S16.** The pathway tool WebGestalt identifies significantly (FDR<0.05) overrepresented Gene Ontology (GO) biological processes within the 36 age-DE ASPC marker genes with consistent DE across ASPCs from the SAT and VAT fat depots (see the excel file Additional file 3: Table S16.xlsx). **Table S17.** Seventy-five of the 76 age-DE SAT ASPC genes are DE across the 6 measured timepoints during a 14-day SAT ASPC differentiation (i.e. adipogenesis) experiment (see the excel file Additional file 3: Table S17.xlsx). **Table S18.** Clustering of the age-DE SAT ASPC genes by their expression profiles during a 14-day SAT ASPC differentiation (i.e. adipogenesis) experiment (see the excel file Additional file 3: Table S18.xlsx). **Table S19.** Gene set enrichment analysis using EnrichR and the human subset of the ChIP Enrichment Analysis (CHEA) 2022 database identifies 5 subgroups significantly enriched for common transcription factor targets. **Table S20.** Intercellular interactions involving the 76 age-DE SAT ASPC genes are significantly stronger (*p*<0.05) in the older individuals of the adipose snRNA-seq cohort compared to the younger individuals. **Table S21.** Interactions involving the gene *RORA*show the largest differences in strength between the younger and older individuals (see the excel file Additional file 3: Table S21.xlsx). **Table S22.** Variance in body mass index (BMI) explained by the genome-wide and regional polygenic risk scores (PRSs). **Table S23.** Estimated effect of the interaction between the age-DE regional BMI PRS and age on BMI in the individuals with normal BMI (BMI<25) and in the obese individuals (BMI>=30) in the UK Biobank. **Table S24.** Local variants within the age-DE SAT ASPC genes landing in ASPC open chromatin regions interact with age on BMI in obese individuals of the UK Biobank (*n*=45,203).

**Additional file 3:** **Table S5.** ASPC marker genes from SAT snRNA-seq data of 6 individuals in FTC. **Table S8.** Seventy-six SAT ASPC genes (i.e. 72 ASPC marker genes and four transcription factor genes regulating ASPC marker genes) are significantly differentially expressed (DE) by age (FDR<0.05) using the ASPC data from the SAT snRNA-seq cohort (*n*=15). **Table S11.** The pathway tool WebGestalt identifies significantly (FDR<0.05) overrepresented developmental and differentiation-related Gene Ontology biological processes within the 76 age-DE SAT ASPC genes. **Table S12.** The pathway tool WebGestalt identifies significantly (FDR<0.05) overrepresented Gene Ontology biological processes within the 79 non-age-DE SAT ASPC genes. **Table S13.** Significant correlations (FDR<0.05) between the expression of the 76 age-DE SAT ASPC genes and cardiometabolic phenotypes using subcutaneous adipose bulk RNA-seq data from METSIM (*n*=335). **Table S14.** Significant correlations (FDR<0.05) between the expression of the 79 non-age-DE SAT ASPC marker genes and cardiometabolic phenotypes using subcutaneous adipose bulk RNA-seq data from METSIM (*n*=335). **Table S15.** Nearly half (47.4%) of the 76 age-DE SAT ASPC genes show significant DE by age (FDR<0.05 in the same direction as in the SAT snRNA-seq cohort) in VAT ASPCs from a publicly available VAT snRNA-seq data of 10 individuals. **Table S16.** The pathway tool WebGestalt identifies significantly (FDR<0.05) overrepresented Gene Ontology (GO) biological processes within the 36 age-DE ASPC marker genes with consistent DE across ASPCs from the SAT and VAT fat depots. **Table S17.** Seventy-five of the 76 age-DE SAT ASPC genes are all DE across the 6 measured timepoints during a 14-day SAT ASPC differentiation (i.e. adipogenesis) experiment. **Table S18.** Clustering of the age-DE SAT ASPC genes by their expression profiles during a 14-day SAT ASPC differentiation (i.e. adipogenesis) experiment.

## Data Availability

The data that support the findings in this manuscript are available from the UK Biobank. However, restrictions apply to the availability of these data, which were used in this study under UK Biobank Application number 33934. UK Biobank data are available for bona fide researchers through the application process: https://www.ukbiobank.ac.uk/learn-more-about-uk-biobank/contact-us. The existing VAT snRNA-seq data from Emont et al. [56] were deposited at Single Cell Portal, under study number SCP1376, and are available for downloading at: https://singlecell.broadinstitute.org/single_cell/study/SCP1376. The existing SAT snRNA-seq data from the FTC [23–26] and in CRYO [38, 50, 51] cohorts were previously made available in the NIH Gene Expression Omnibus (GEO), under accession number GSE236708 (https://www.ncbi.nlm.nih.gov/geo/query/acc.cgi?acc=GSE236708) [49]. The existing SAT snRNA-seq data on the six Finnish twin samples have been deposited into the THL Biobank, Helsinki, Finland, under accession number THLBB2020_PUB002: https://thl.fi/en/research-and-development/thl-biobank/for-researchers/sample-collections/twin-study. There is an application process to follow for researchers: https://thl.fi/en/research-and-development/thl-biobank/for-researchers/application-process. The existing ASPC ATAC-seq data in FTC from Garske et al. [59] were previously made available in GEO, under accession number GSE235363 (https://www.ncbi.nlm.nih.gov/geo/query/acc.cgi?acc=GSE235363). The existing METSIM bulk SAT RNA-seq data from Pan et al. [46] were previously made available in GEO, under accession number GSE135134 (https://www.ncbi.nlm.nih.gov/geo/query/acc.cgi?acc=GSE135134) [47]. The METSIM SAT snRNA-seq data are available in GEO, under accession number GSE249089 (https://www.ncbi.nlm.nih.gov/geo/query/acc.cgi?acc=GSE249089) [44]. The bulk RNA-seq data from the primary human preadipocyte differentiation experiment are available in GEO, under accession number GSE249195 (https://www.ncbi.nlm.nih.gov/geo/query/acc.cgi?acc=GSE249195) [65].

## Abbreviations

ALT: Alanine aminotransferase
ASPC: Adipose stem and precursor cell
ATAC: Assay for transposase accessible chromatin
BMI: Body mass index
CCA: Canonical correlation analysis
CRP: C-reactive protein
DE: Differential expression
FTC: Finnish Twin Cohort
GWAS: Genome-wide association study
HOMAIR: Homeostatic Model Assessment for Insulin Resistance
HOMER: Hypergeometric Optimization of Motif EnRichment
HuGE: Human Genetic Evidence
IL1RA: Interleukin-1 receptor antagonist
IL1b: Interleukin-1 beta
LD: Linkage disequilibrium
METSIM: METabolic Syndrome In Men
MHO: Metabolically healthy obesity
MUO: Metabolically unhealthy obesity
MZ: Monozygotic
PCA: Principal component analysis
PRS: Polygenic risk score
RORA: RAR Related Orphan Receptor A
RNA-seq: RNA sequencing
SAT: Subcutaneous adipose tissue
SMAD3: SMAD Family Member 3
snRNA-seq: Single-nucleus RNA sequencing
T2D: Type 2 diabetes
TF: Transcription factor
TPM: Transcript per million
TWIST2: Twist Family bHLH Transcription Factor 2
UKB: UK Biobank
VAT: Visceral adipose tissue
WHR: Waist-to-hip ratio
WHRadjBMI: Waist-to-hip ratio adjusted for BMI
