# Supplementary material for: Age-dependent genes in adipose stem and precursor cells affect regulation of fat cell differentiation and link aging to obesity via cellular and genetic interactions
Source: Genome Med. 2024 Jan 31;16:19. doi: 10.1186/s13073-024-01291-x (PMC10829214; doi:10.1186/s13073-024-01291-x)
Supplement: Supplementary file 1 — Additional file 1: Fig. S1. Estimated SAT cell-type proportions in the Finnish Twin Cohort (FTC) differ by age, BMI status, and sex. (a) Boxplots compare the scaled cell-type proportion estimates in bulk SAT RNA-seq data from FTC by the BMI status (nlower BMI=50, nhigher BMI=50). The lower BMI twin per pair was classified as lower BMI for the pair and the higher BMI twin as higher BMI for the pair. (b) Boxplots compare the scaled cell-type proportion estimates from FTC between males (n=46) and females (n=54). (c,d) Boxplots compare the SAT ASPC proportions by age within the BMI status groups in (c) females (nbelow 40=34, nover 40=20) and (d) males (nbelow 40=22, nover 40=24) from FTC. (a-d) Asterisks denote a significant difference in cell-type proportions between the colored groups as assessed by a Wilcoxon test. Significance thresholds for p-values:*p <0.05, **p <0.01, and ***p<0.001. Fig. S2. The 76 age-DE SAT ASPC genes contain protein interaction networks with significant enrichments for age, metabolic outcomes, and adipose tissue development. (a,b) The inter-gene relations between (a) the 76 age-DE SAT ASPC genes and (b) 61 age-upregulated SAT ASPC genes are visualized as a knowledge graph. (a, b) Each node indicates a gene, and each edge indicates a relation connecting a set of genes, where edges are colored by relation type as follows: from a curated database (cyan), experimentally determined (magenta), gene cooccurrence (blue), co-expression (black), protein homology (lavender). (b) We shade the gene nodes in networks significantly enriched for key pathways and phenotypes of interest as follows: body weights and measures (blue), age (red), lipid or lipoprotein measurement (green), abnormal glucose homeostasis (yellow), regulation of fat cell differentiation (magenta). The enrichment results are reported in the panel. Fig. S3. Clustering of the protein interaction network induced by the upregulated age-DE SAT ASPC genes identifies six subnetworks of inter-ge [file 13073_2024_1291_MOESM1_ESM.pdf]

Supplementary Figures

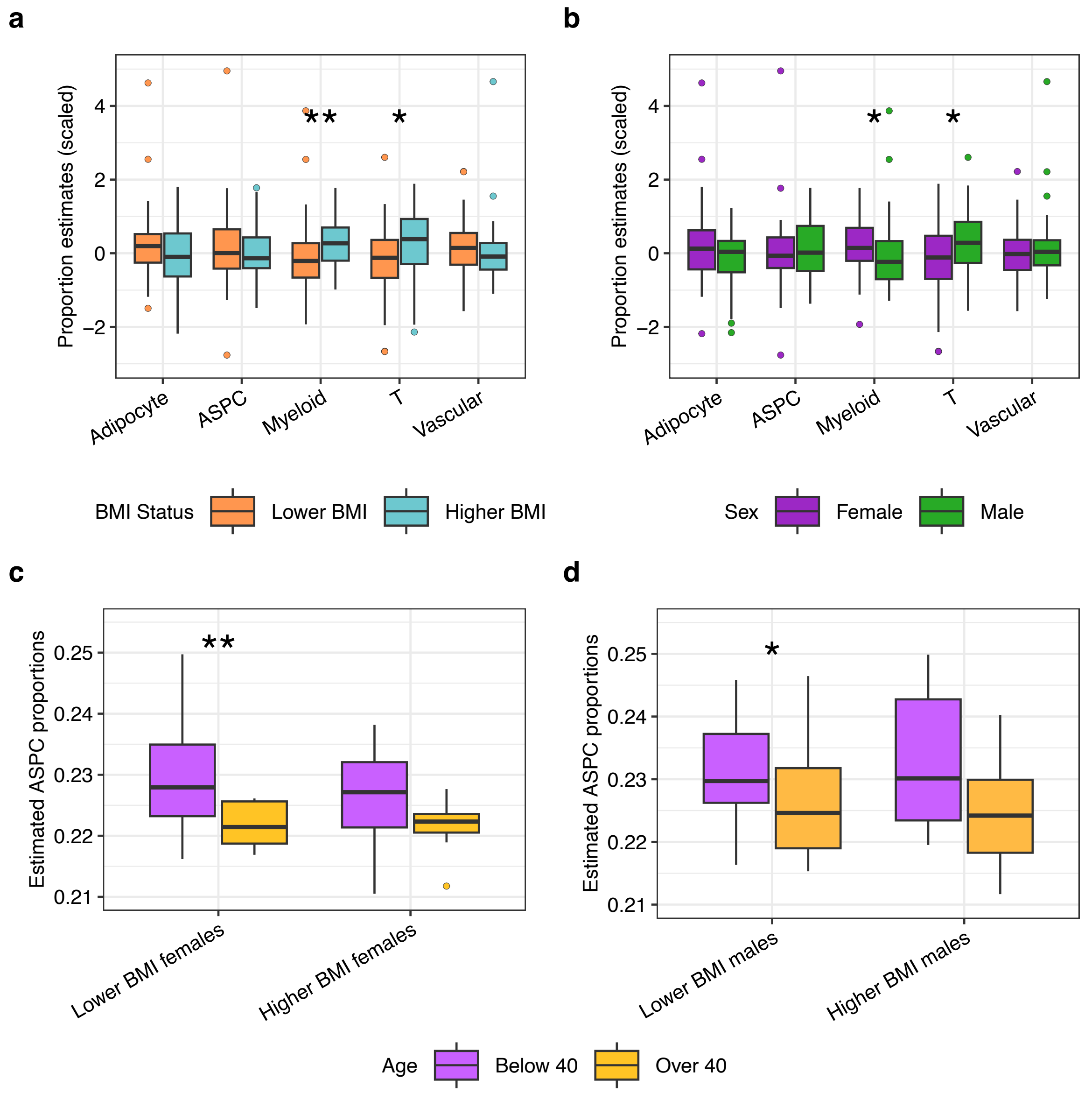

Fig. S1. Estimated SAT cell-type proportions in the Finnish Twin Cohort (FTC) differ by age, BMI status, and sex. (a) Boxplots compare the scaled cell-type proportion estimates in bulk SAT RNA-seq data from FTC by the BMI status ($n_{\text{lower BMI}}$=50, $n_{\text{higher BMI}}$=50). The lower BMI twin per pair was classified as lower BMI for the pair and the higher BMI twin as higher BMI for the pair. (b) Boxplots compare the scaled cell-type proportion estimates from FTC

between males (n=46) and females (n=54). (c,d) Boxplots compare the SAT ASPC proportions by age within the BMI status groups in (c) females ($n_{below\ 40}$=34, $n_{over\ 40}$=20) and (d) males ($n_{below\ 40}$=22, $n_{over\ 40}$=24) from FTC. (a-d) Asterisks denote a significant difference in cell-type proportions between the colored groups as assessed by a Wilcoxon test. Significance thresholds for p-values: *p <0.05, **p <0.01, and ***p <0.001.

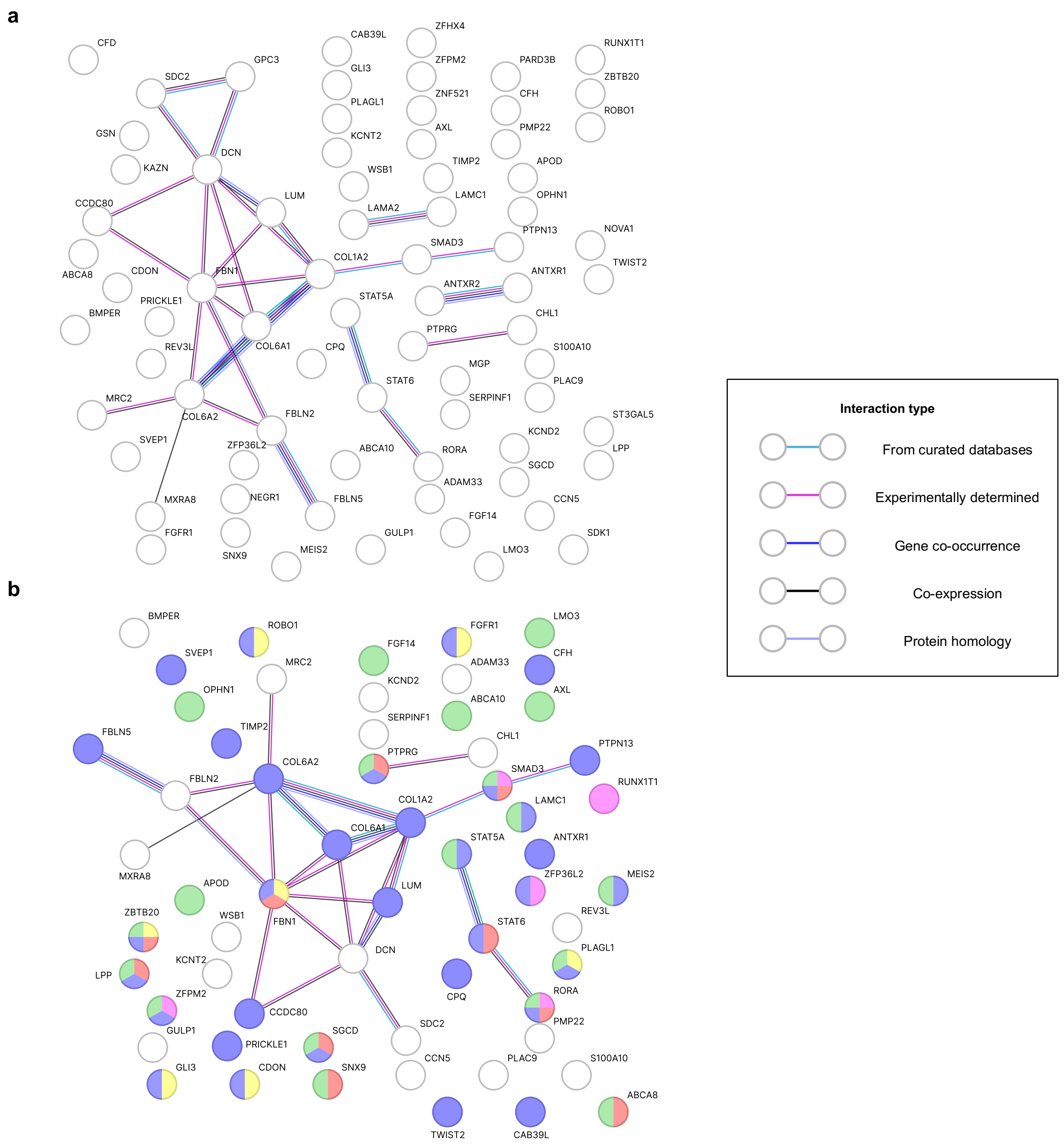

	Category	Term ID	Term description	Observed gene count	FDR
	Monarch	EFO:0004324	Body weights and measures	33	1.64x10^{-5}
	Monarch	EFO:0000246	Age	10	4.90x10^{-3}
	Monarch	EFO:0005105	Lipid or lipoprotein measurement	19	6.40x10^{-3}
	Monarch	HP:0011014	Abnormal glucose homeostasis	7	0.0319
	GO Process	GO:0045598	Regulation of fat cell differentiation	5	7.50x10^{-3}

Fig. S2. The 76 age-DE SAT ASPC genes contain protein interaction networks with

significant enrichments for age, metabolic outcomes, and adipose tissue development. (a,b) The inter-gene relations between (a) the 76 age-DE SAT ASPC genes and (b) 61 age-upregulated SAT ASPC genes are visualized as a knowledge graph. (a, b) Each node indicates a gene, and each edge indicates a relation connecting a set of genes, where edges are colored by relation type as follows: from a curated database (cyan), experimentally determined (magenta), gene co-occurrence (blue), co-expression (black), protein homology (lavender). (b) We shade the gene nodes in networks significantly enriched for key pathways and phenotypes of interest as follows: body weights and measures (blue), age (red), lipid or lipoprotein measurement (green), abnormal glucose homeostasis (yellow), regulation of fat cell differentiation (magenta). The enrichment results are reported in the panel.

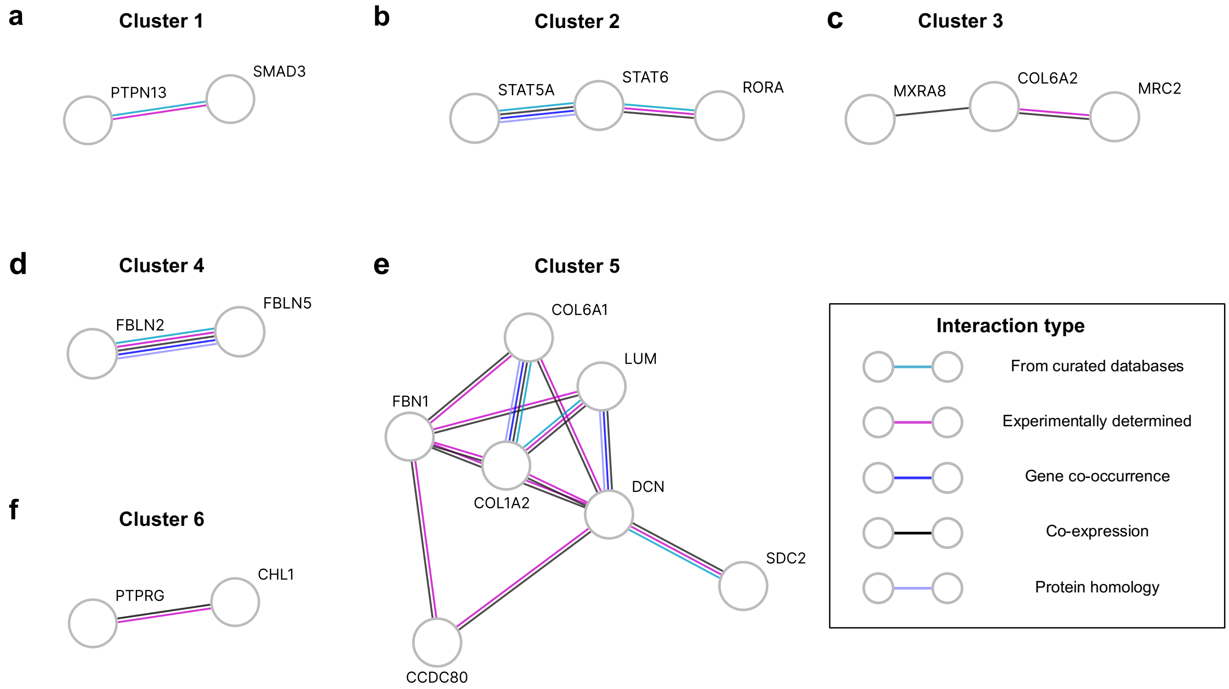

Fig. S3. Clustering of the protein interaction network induced by the upregulated age-DE SAT ASPC genes identifies six subnetworks of inter-gene relations. (a-f) The clusters derived from the protein network of the 61 age-upregulated SAT ASPC genes are visualized as individual knowledge graphs. Each node indicates a gene, and each edge indicates a relation connecting a set of genes, where edges are colored by relation type as follows: from a curated database (cyan), experimentally determined (magenta), gene co-occurrence (blue), co-expression (black), protein homology (lavender).

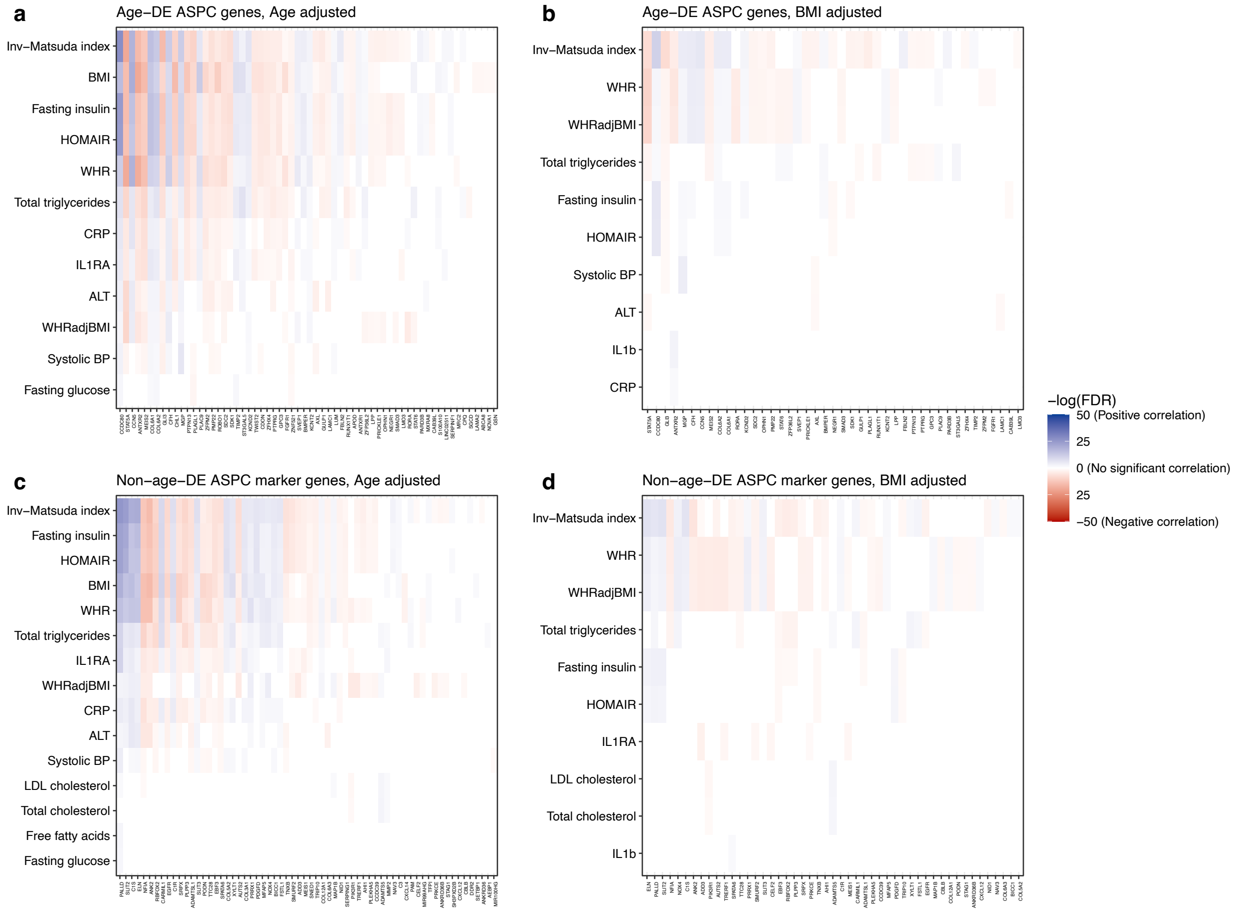

Gene set	BMI	HOMAIR	Inv−Matsuda index	CRP	Free fatty acids	Fasting glucose	IL1RA	Fasting insulin	ALT	LDL cholesterol	Total cholesterol	Total triglycerides	Systolic BP	WHR	WHRadjBMI	IL1b
% Correlated in Non−age−DE Age adjusted	54.43%	58.23%	68.35%	35.44%	1.27%	1.27%	40.51%	56.96%	25.32%	7.59%	3.80%	48.10%	21.52%	60.76%	43.04%	0.00%
% Correlated in Age−DE Age adjusted	67.11%	67.11%	71.05%	46.05%	0.00%	7.89%	44.74%	67.11%	31.58%	0.00%	0.00%	53.95%	22.37%	57.89%	31.58%	0.00%
% Correlated in Non−age−DE BMI adjusted	NA	8.86%	43.04%	0.00%	0.00%	0.00%	7.59%	12.66%	0.00%	2.53%	2.53%	16.46%	0.00%	40.51%	40.51%	1.27%
% Correlated in Age−DE BMI adjusted	NA	7.89%	40.79%	1.32%	0.00%	0.00%	0.00%	14.47%	3.95%	0.00%	0.00%	19.74%	3.95%	32.89%	28.95%	1.32%

Fig. S4. Age and BMI influence association patterns between metabolic phenotypes and the bulk expression of both the age-DE ASPC genes and non-age-DE ASPC marker genes in the bulk SAT RNA-seq data from the METSIM cohort (n=335). Heatmaps display significant associations (FDR<0.05) between metabolic outcomes and the bulk expression of (a, b) the 76 age-DE ASPC genes and (c, d) the 79 non-age-DE ASPC marker genes. Each outcome has been adjusted for (a, c) age or (b, d) BMI. (a-d) Gene-outcome pairs are color-coded based

on directionality and significance: red indicates a positive correlation, blue indicates a negative correlation, and white represents no significant correlation. Significance was assessed by a Wilcoxon rank sum test and a positive log_2 fold change in gene expression with the outcome was defined as a positive association. We excluded genes and outcomes not identified in any significant gene-outcome pair. Below the panels, a table summarizes the proportions of genes per gene set that show significant associations with each adjusted outcome. Abbreviations are as follows: BMI indicates body mass index; HOMAIR Homeostatic Model Assessment for Insulin Resistance; C-reactive protein; IL1RA interleukin-1 receptor antagonist; ALT alanine transaminase; WHR waist-to-hip ratio; WHRadjBMI waist-to-hip ratio adjusted for BMI; and IL1b Interleukin-1 beta.

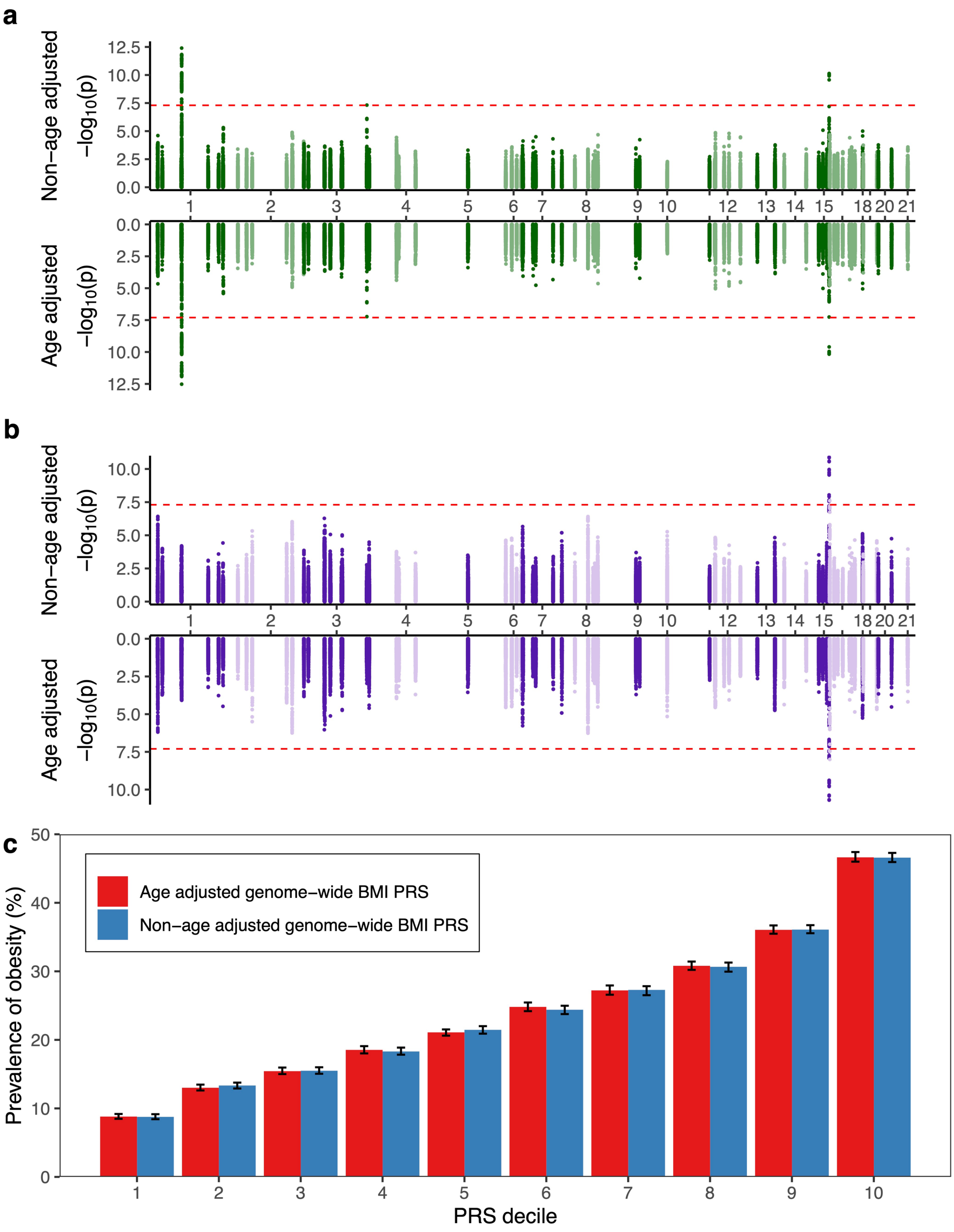

Fig. S5. Age adjustments minimally alter the results of BMI GWASs and predictive

performance of BMI PRSs in the UK Biobank. (a,b) A Miamiplot visualizes the effect of age adjustment on the summary level results of BMI GWAS using unrelated European individuals from UKB. Variants in the *cis*-regions of the age-DE ASPC genes are plotted by chromosomal position against the $-\log_{10}$(p-value) from BMI GWASs conducted with (a) only males (n=90,045) and (c) only females (n=105,818). The bottom panel reports the BMI GWAS where age and age^2 were included as covariates, while in the BMI GWAS for the top panel, no age-related term was used as a covariate. A horizontal line marks the genome-wide significance threshold ($p=5x10^{-8}$). (c) Paired bar plots compare the proportion of obese individuals (BMI>=30) in each PRS decile between the age-adjusted and non-age adjusted genome-wide BMI PRSs created using unrelated European individuals from the UKB (n=193,602). The error bars represent the 95% confidence intervals of the proportions.

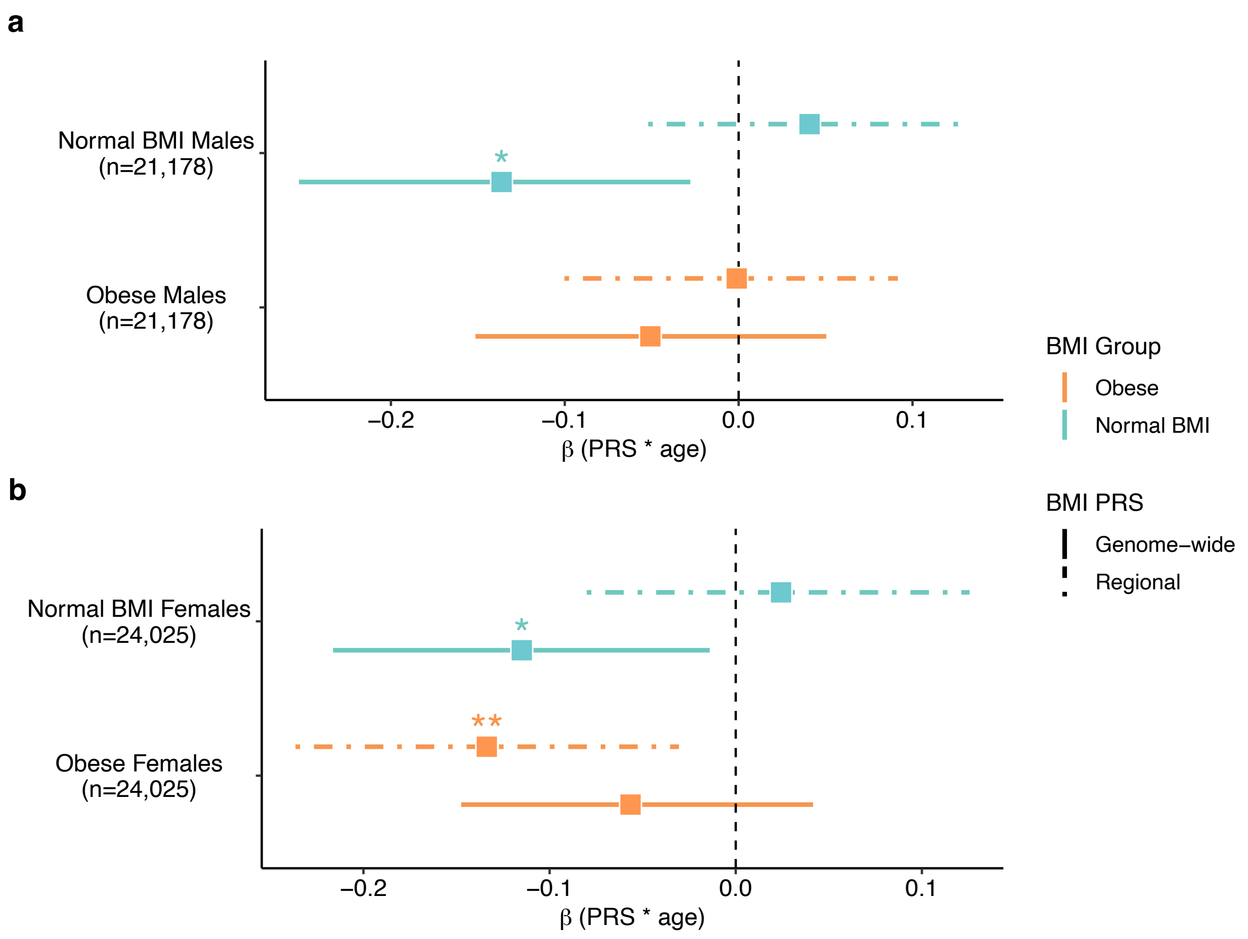

Fig. S6. Sex-stratified PRSs identify sex-specific effects on BMI and on age-obesity interactions in the *cis*-regions of the age-DE ASPC genes in the UK Biobank. (a,b) Forest plots compare the 95% confidence intervals for the standardized estimated coefficient (β) of the age and BMI PRS interaction term using the regional and genome-wide PRSs constructed for the (a) males (n=88,988) and (b) females (n=104,614) in UKB. We separated the normal BMI (BMI<25) and obese (BMI>=30) individuals in each sex. Asterisks indicate that the age and BMI PRS interaction term is significant in the model, as assessed by a Wald-test. Significance thresholds for p-values: *p <0.05, **p <0.01, and ***p <0.001.

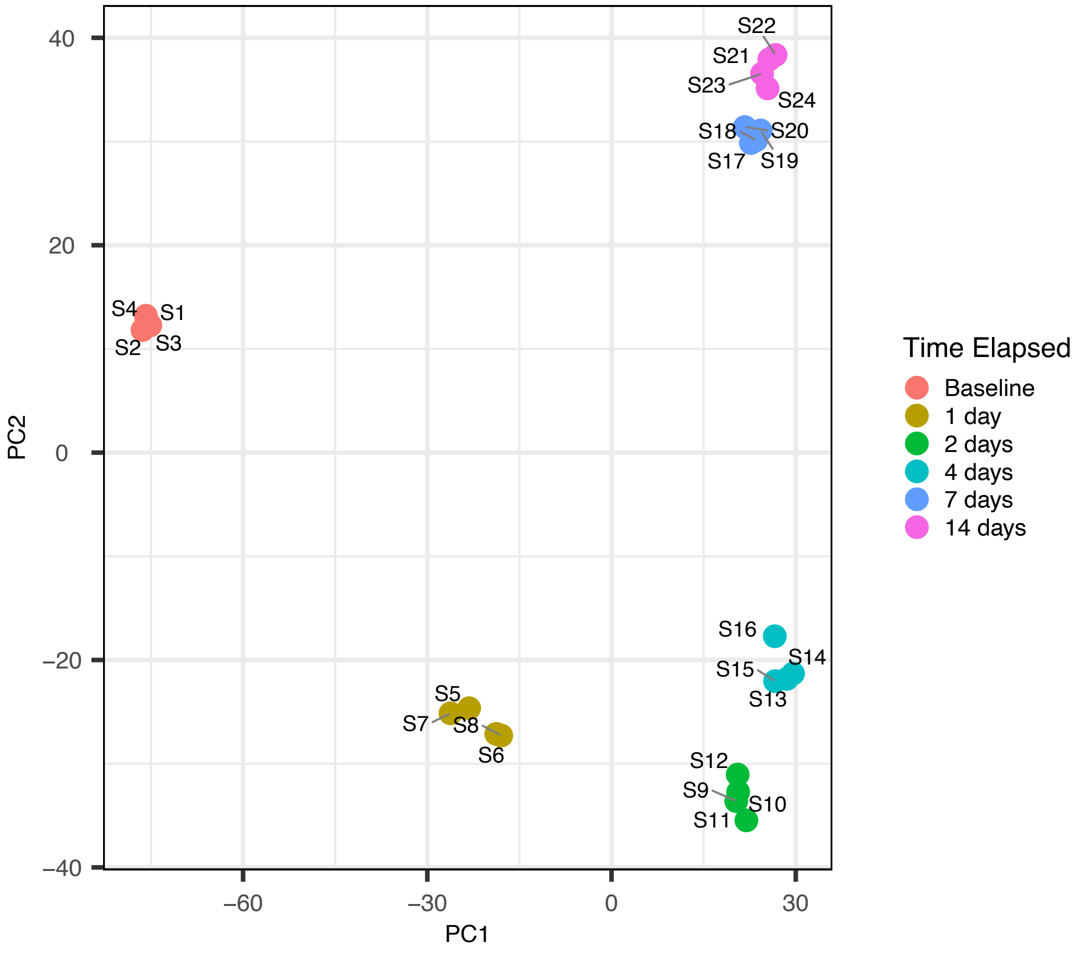

Fig. S7. PCA analysis of SAT adipogenesis time point data shows clustering by the time-point. Biplot compares the first and second principal component (PC) of the expression data of each sample from the SAT ASPC differentiation experiment ($n_{samples}$=24, i.e. one sample in 4 isogenic biological replicates in six time points). We colored points by the time elapsed between the start of the experiment and when the sample was taken. PCs were calculated using the log_2-transformed transcripts per million.
