# Supplementary material for: Age-dependent genes in adipose stem and precursor cells affect regulation of fat cell differentiation and link aging to obesity via cellular and genetic interactions
Source: Genome Med. 2024 Jan 31;16:19. doi: 10.1186/s13073-024-01291-x (PMC10829214; doi:10.1186/s13073-024-01291-x)
Supplement: Supplementary file 2 — Additional file 2: Table S1. Summary of the estimated proportions of the 5 major subcutaneous adipose tissue cell-types in FTC (n=100). Table S2. Wilcoxon tests comparing proportions of the 5 major subcutaneous adipose tissue cell-types between the unrelated individuals below (n=28) and above 40 years old (n=22) in FTC. Table S3. Summary of the estimated proportions of the 20 major subcutaneous adipose tissue subcell-types/cell-types in METSIM (n=335). Table S4. Down sampling the numbers of individuals to match sample sizes between the younger and older groups preserves the observed SAT ASPC proportion differences in FTC and METSIM (see Table 1). Table S5. ASPC marker genes from SAT snRNA-seq data of 6 individuals in FTC (see the excel file Additional file 3: Table S5.xlsx). Table S6. Known motifs from HOMER enriched in promoter regions of the 151 unique SAT ASPC marker genes. Table S7. Motifs determined by de novo motif discovery in HOMER to be significantly enriched (p<1x10^-10^) in the promoter regions of the 151 unique SAT ASPC marker genes. Table S8. Seventy-six SAT ASPC genes (i.e. 72 ASPC marker genes and four transcription factor genes regulating ASPC marker genes) are significantly differentially expressed (DE) by age (FDR<0.05) using the ASPC data from the SAT snRNA-seq cohort (n=15) (see the excel file Additional file 3: Table S8.xlsx). Table S9. Our additional analysis in the independent METSIM cohort shows that 41% of the 76 age-DE SAT ASPC genes exhibit consistent DE (FDR<0.1 and the same direction as in the discovery snRNA-seq cohort) in the ASPCs from the subcutaneous adipose snRNA-seq data of the youngest and oldest age quartiles of the METSIM cohort. Table S10. Our additional analysis in the independent METSIM cohort shows that 49% of the 76 age-DE SAT ASPC genes exhibit consistent DE (FDR<0.1 and the same direction of effect as in the discovery cohort) in the ASPCs from the subcutaneous adipose snRNA-seq data of nonobese (BMI<30) individuals from th [file 13073_2024_1291_MOESM2_ESM.pdf]

Supplementary Tables

Table S1. Summary of the estimated proportions of the 5 major subcutaneous adipose tissue cell-types in FTC (n=100).

Cell-type	Minimum	1st quartile	Median	Mean	3rd quartile	Maximum	Standard deviation
ASPC	0.167	0.220	0.225	0.226	0.232	0.293	0.013
Vascular	0.056	0.154	0.162	0.162	0.168	0.246	0.018
Myeloid	0.064	0.131	0.137	0.138	0.146	0.192	0.014
Adipocyte	0.322	0.410	0.427	0.425	0.440	0.558	0.029
T	0.011	0.041	0.049	0.049	0.058	0.086	0.014

Table S2. Wilcoxon tests comparing proportions of the 5 major subcutaneous adipose tissue cell-types between the unrelated individuals below (n=28) and above 40 years old (n=22) in FTC.

Cell-type	Mean ± SD of below 40[A]	Mean ± SD of over 40[B]	W statistic[C]	p-value[D]
ASPC	0.229 ± 0.011	0.218 ± 0.015	454	0.0038
Vascular	0.166 ± 0.012	0.153 ± 0.023	453	0.0004
Myeloid	0.135 ± 0.009	0.142 ± 0.013	200	0.0348
Adipocyte	0.422 ± 0.024	0.437 ± 0.035	219	0.0837
T	0.048 ± 0.016	0.050 ± 0.013	295	0.8087

[A] Mean ± standard deviation of the adipose cell-type proportions in the individuals with age below 40 years.

[B] Mean ± standard deviation of the adipose cell-type proportions in the individuals with age over 40 years.

[C] Wilcoxon test statistic between the adipose cell-type proportions in the individuals below and above 40 years old.

[D] Wilcoxon test p-value between the adipose cell-type proportions in the individuals below and above 40 years old.

Table S3. Summary of the estimated proportions of the 20 major subcutaneous adipose tissue subcell-types/cell-types in METSIM (n=335).

Cell-type	Minimum	1st quartile	Median	Mean	3rd quartile	Maximum	Standard deviation
T_1	0.010	0.121	0.126	0.130	0.138	0.297	0.034
T_2	0.020	0.106	0.115	0.115	0.122	0.380	0.037
Adip_1	0.007	0.113	0.118	0.118	0.121	0.501	0.043
T_3	0	0.056	0.062	0.067	0.074	0.239	0.028
T_4	0	0.046	0.049	0.049	0.052	0.210	0.021
Myel_1	0.009	0.058	0.061	0.061	0.063	0.152	0.015
Vasc_1	0.008	0.059	0.063	0.065	0.070	0.212	0.022
Adip_2	0.003	0.055	0.058	0.059	0.061	0.202	0.020
Myel_2	0	0.035	0.042	0.041	0.046	0.129	0.015
B	0	0.032	0.035	0.034	0.036	0.109	0.012
Myel_3	0.002	0.042	0.048	0.049	0.054	0.181	0.022
Myel_4	0	0.021	0.022	0.023	0.025	0.064	0.008
Vasc_2	0	0.023	0.025	0.024	0.026	0.071	0.008
Vasc_3	0	0.015	0.023	0.023	0.029	0.111	0.013
Ntrphl	0	0.012	0.014	0.015	0.016	0.156	0.015
Myel_5	0	0.004	0.008	0.008	0.010	0.035	0.005
Ribo	0	0.003	0.006	0.005	0.007	0.061	0.006
Mast	0	0	0.002	0.002	0.004	0.017	0.003
ASPC	0.012	0.086	0.091	0.092	0.097	0.248	0.024
Fibr	0	0.018	0.020	0.020	0.022	0.100	0.009

Table S4. Down sampling the numbers of individuals to match sample sizes between the younger and older groups preserves the observed SAT ASPC proportion differences in FTC and METSIM (see Table 1).

**Cohort**[A]	**Group**[B]	N_{young}[C]	N_{old}[D]	**Mean ASPC proportion ± SD**[E] **of young**	**Mean ASPC proportion ± SD of old**	**W statistic**[F]	**p-value**[G]
FTC	Lower BMI	22	22	0.231 ± 0.009	0.227 ± 0.011	348	0.0122
FTC	Higher BMI	22	22	0.228 ± 0.011	0.220 ± 0.014	319	0.0723
METSIM	Normal BMI	18	18	0.096 ± 0.008	0.089 ± 0.006	238	0.0159
METSIM	Overweight	18	18	0.096 ± 0.007	0.090 ± 0.008	189	0.4064
METSIM	Obese	18	18	0.089 ± 0.006	0.092 ± 0.006	127	0.2788

[A] Finnish Twin Cohort (FTC) or METSIM cohort

[B] The BMI status group used for comparisons of ASPC proportions by age

[C] Number of individuals in the younger group

[D] Number of individuals in the older group

[E] Mean ± standard deviation (SD) of ASPC proportions

[F] Wilcoxon test statistic between the ASPC proportions in the young and old individuals

[G] Wilcoxon test p-value between the ASPC proportions in the young and old individuals

Table S5. ASPC marker genes from SAT snRNA-seq data of 6 individuals in FTC (see the excel file Additional File 3: Table S5.xlsx).

Table S6. Known motifs from HOMER enriched in promoter regions of the 151 unique SAT ASPC marker genes.

Motif Name	p-value[A]
TBP	0.001
CEBPB	0.001
SMAD3	0.01
FOXA1	0.01
STAT5A	0.01
CDX2	0.01
ZNF652	0.01
TWIST2	0.01
SIX4	0.01
NEUROG2	0.01
SOX9	0.01
NFAT	0.01
MYOD1	0.01
STAT6	0.01
KLF10	0.01
HLF	0.01
OCT11 (POU2F3)	0.01
NFIL3	0.01

[A] As determined by HOMER.

Table S7. Motifs determined by *de novo* motif discovery in HOMER to be significantly enriched ($p<1x10^{-10}$) in the promoter regions of the 151 unique SAT ASPC marker genes.

Motif name	p-value[A]
PRDM1	$1x10^{-15}$
MAFK	$1x10^{-12}$
TCF3	$1x10^{-11}$

[A]As determined by HOMER.

Table S8. Seventy-six SAT ASPC genes (i.e. 72 ASPC marker genes and four transcription factor genes regulating ASPC marker genes) are significantly differentially expressed (DE) by age (FDR<0.05) using the ASPC data from the SAT snRNA-seq cohort (n=15) (see the excel file Additional File 3: Table S8.xlsx).

Table S9. Our additional analysis in the independent METSIM cohort shows that 41% of the 76 age-DE SAT ASPC genes exhibit consistent DE (FDR<0.1 and the same direction as in the discovery snRNA-seq cohort) in the ASPCs from the subcutaneous adipose snRNA-seq data of the youngest and oldest age quartiles of the METSIM cohort.

Gene name	log_2FC^A	p-value	FDR	Same Direction as Original[B]
CHL1	0.447	1.25E-08	9.28E-07	TRUE
PLAGL1	0.349	5.50E-08	2.03E-06	FALSE
ZFPM2	0.160	4.91E-06	1.20E-04	TRUE
LMO3	0.176	6.56E-06	1.20E-04	TRUE
GLI3	0.146	8.12E-06	1.20E-04	FALSE
SNX9	0.288	1.51E-05	1.86E-04	TRUE
PARD3B	0.240	2.19E-05	2.32E-04	TRUE
S100A10	0.496	2.81E-05	2.60E-04	TRUE
RORA	0.164	3.30E-05	2.71E-04	TRUE
LPP	0.119	4.74E-05	3.51E-04	TRUE
KCND2	0.059	1.47E-04	9.13E-04	FALSE
OPHN1	0.036	1.48E-04	9.13E-04	FALSE
COL1A2	0.031	2.72E-04	1.48E-03	FALSE
LAMC1	-0.008	2.89E-04	1.48E-03	FALSE
GULP1	0.021	3.01E-04	1.48E-03	TRUE
ABCA8	0.047	3.74E-04	1.73E-03	TRUE
FGF14	0.245	5.42E-04	2.36E-03	TRUE
MEIS2	0.088	7.08E-04	2.83E-03	FALSE
CFD	0.066	7.27E-04	2.83E-03	TRUE
GPC3	-0.013	8.66E-04	3.20E-03	FALSE
FBN1	0.006	1.04E-03	3.68E-03	TRUE
SVEP1	0.212	1.47E-03	4.94E-03	FALSE
TWIST2	0.171	1.62E-03	5.20E-03	FALSE
DCN	-0.035	2.66E-03	8.19E-03	FALSE
APOD	-0.085	3.05E-03	9.03E-03	FALSE
KAZN	0.127	4.10E-03	0.012	TRUE
ZFHX4	-0.007	4.86E-03	0.013	FALSE
COL6A2	0.017	5.33E-03	0.014	TRUE
NEGR1	0.132	5.70E-03	0.014	TRUE
ZBTB20	0.071	5.84E-03	0.014	TRUE
COL6A1	-0.092	6.18E-03	0.015	FALSE
PRICKLE1	0.389	6.51E-03	0.015	TRUE

CDON	-0.018	6.55E-03	0.015	TRUE
ADAM33	0.078	6.70E-03	0.015	TRUE
SDK1	0.013	7.73E-03	0.016	TRUE
SGCD	0.261	9.59E-03	0.020	TRUE
ST3GAL5	0.207	0.012	0.024	TRUE
ROBO1	-0.076	0.013	0.025	FALSE
BMPER	0.240	0.016	0.030	TRUE
ANTXR2	-0.016	0.016	0.030	FALSE
FBLN5	0.142	0.018	0.032	TRUE
CPQ	-0.043	0.019	0.033	FALSE
TIMP2	-0.021	0.019	0.033	TRUE
SMAD3	-0.014	0.020	0.034	TRUE
SERPINF1	0.124	0.029	0.047	TRUE
ANTXR1	-0.061	0.030	0.047	FALSE
ABCA10	0.032	3.97E-02	6.25E-02	TRUE
CCDC80	-0.207	4.75E-02	7.33E-02	TRUE
MGP	0.168	5.18E-02	7.82E-02	TRUE
ABCA9-AS1	-0.054	5.34E-02	7.85E-02	FALSE
FBLN2	-0.239	5.41E-02	7.85E-02	FALSE
MRC2	-0.018	6.58E-02	9.36E-02	FALSE

[A]Average log_2 fold change in the expression in ASPCs between the individuals in the lowest age quartile (age<=51, n=21) and highest age quartile (age>58, n=21). A positive log_2 fold change represents greater expression in the older age quartile.

[B] TRUE indicates that the direction of the expression change with age is consistent with the adipose snRNA-seq cohort (Additional File 3: Table S8).

Table S10. Our additional analysis in the independent METSIM cohort shows that 49% of the 76 age-DE SAT ASPC genes exhibit consistent DE (FDR<0.1 and the same direction of effect as in the discovery cohort) in the ASPCs from the subcutaneous adipose snRNA-seq data of non-obese (BMI<30) individuals from the METSIM cohort (n=38).

Gene name	log_2FC[A]	p-value	FDR	Same Direction as Original[B]
PLAGL1	0.342	8.00E-09	5.92E-07	FALSE
CHL1	0.390	1.88E-08	6.94E-07	TRUE
LMO3	0.289	5.71E-07	1.41E-05	TRUE
RORA	0.219	1.07E-06	1.99E-05	TRUE
ZFPM2	0.177	1.46E-06	2.16E-05	TRUE
SNX9	0.332	2.09E-06	2.58E-05	TRUE
PARD3B	0.258	5.18E-06	5.48E-05	TRUE
S100A10	0.472	7.20E-06	6.66E-05	TRUE
GULP1	0.205	8.21E-06	6.75E-05	TRUE
GLI3	0.091	1.44E-05	1.06E-04	FALSE
LPP	0.140	3.47E-05	2.27E-04	TRUE
KCND2	0.126	3.68E-05	2.27E-04	FALSE
OPHN1	0.041	5.92E-05	3.37E-04	FALSE
GPC3	0.082	8.07E-05	4.26E-04	TRUE
COL1A2	0.014	1.05E-04	4.95E-04	FALSE
MEIS2	0.062	1.07E-04	4.95E-04	FALSE
ABCA8	0.067	1.17E-04	5.08E-04	TRUE
CFD	0.060	1.36E-04	5.33E-04	TRUE
FBN1	0.037	1.37E-04	5.33E-04	TRUE
LAMC1	-0.050	1.95E-04	7.22E-04	FALSE
FGF14	0.253	3.03E-04	1.07E-03	TRUE
CDON	0.093	5.64E-04	1.90E-03	FALSE
KAZN	0.159	6.96E-04	2.24E-03	TRUE
SGCD	0.298	7.66E-04	2.36E-03	TRUE
NEGR1	0.165	9.10E-04	2.61E-03	TRUE
ZBTB20	0.113	9.16E-04	2.61E-03	TRUE
ADAM33	0.186	1.23E-03	3.37E-03	TRUE
CPQ	0.040	1.69E-03	4.39E-03	TRUE
APOD	-0.169	1.72E-03	4.39E-03	FALSE
ZFHX4	0.020	1.98E-03	4.87E-03	TRUE
TWIST2	0.072	2.07E-03	4.95E-03	FALSE
DCN	-0.050	2.20E-03	5.10E-03	FALSE

FBLN5	0.242	2.52E-03	5.49E-03	TRUE
SVEP1	0.182	2.50E-03	5.49E-03	FALSE
COL6A1	-0.120	2.79E-03	5.90E-03	FALSE
SDK1	0.162	3.75E-03	7.69E-03	TRUE
PRICKLE1	0.383	3.84E-03	7.69E-03	TRUE
ANTXR2	0.055	4.38E-03	8.32E-03	TRUE
ROBO1	-0.079	4.36E-03	8.32E-03	FALSE
ST3GAL5	0.250	4.88E-03	9.02E-03	TRUE
ANTXR1	-0.046	5.91E-03	1.07E-02	FALSE
BMPER	0.254	6.56E-03	1.16E-02	TRUE
TIMP2	-0.022	9.44E-03	1.62E-02	TRUE
ABCA9-AS1	0.033	1.02E-02	1.72E-02	TRUE
CCDC80	-0.178	1.12E-02	1.81E-02	TRUE
SDC2	0.062	1.13E-02	1.81E-02	TRUE
SERPINF1	0.271	1.20E-02	1.88E-02	TRUE
SMAD3	-0.041	1.28E-02	1.97E-02	TRUE
WSB1	-0.145	1.41E-02	2.13E-02	TRUE
COL6A2	-0.154	1.72E-02	2.54E-02	FALSE
FBLN2	-0.173	2.11E-02	3.06E-02	FALSE
MGP	0.181	2.57E-02	3.65E-02	TRUE
MRC2	-0.015	2.83E-02	3.94E-02	FALSE
REV3L	0.011	4.04E-02	5.54E-02	TRUE
RUNX1T1	-0.119	4.16E-02	5.60E-02	FALSE
ABCA10	0.017	4.54E-02	5.99E-02	TRUE

[A]Average log_2 fold change in expression in ASPCs between the individuals in the lowest age quartile (age<=51, n=19) and highest age quartile (age>58, n=19). A positive log_2 fold change represents greater expression in the older age quartile.

[B] TRUE indicates that the direction of the expression change with age is consistent with the adipose snRNA-seq cohort (Additional File 3: Table S8).

Table S11. The pathway tool WebGestalt identifies significantly (FDR<0.05) overrepresented developmental and differentiation-related Gene Ontology biological processes within the 76 age-DE SAT ASPC genes (see the excel file Additional File 3: Table S11.xlsx).

Table S12. The pathway tool WebGestalt identifies significantly (FDR<0.05) overrepresented Gene Ontology biological processes within the 79 non-age-DE SAT ASPC genes (see the excel file Additional File 3: Table S12.xlsx).

Table S13. Significant correlations (FDR<0.05) between the expression of the 76 age-DE SAT ASPC genes and cardiometabolic phenotypes using subcutaneous adipose bulk RNA-seq data from METSIM (n=335) (see the excel file Additional File 3: Table S13.xlsx).

Table S14. Significant correlations (FDR<0.05) between the expression of the 79 non-age-DE SAT ASPC marker genes and cardiometabolic phenotypes using subcutaneous adipose bulk RNA-seq data from METSIM (n=335) (see the excel file Additional File 3: Table S14.xlsx).

Table S15. Nearly half (47.4%) of the 76 age-DE SAT ASPC genes show significant DE by age (FDR<0.05 in the same direction as in the SAT snRNA-seq cohort) in VAT ASPCs from a publicly available VAT snRNA-seq data of 10 individuals (see the excel file Additional File 3: Table S15.xlsx).

Table S16. The pathway tool WebGestalt identifies significantly (FDR<0.05) overrepresented Gene Ontology (GO) biological processes within the 36 age-DE ASPC marker genes with consistent DE across ASPCs from the SAT and VAT fat depots (see the excel file Additional File 3: Table S16.xlsx).

Table S17. Seventy-five of the 76 age-DE SAT ASPC genes are DE across the 6 measured timepoints during a 14-day SAT ASPC differentiation (i.e. adipogenesis) experiment (see the excel file Additional File 3: Table S17.xlsx).

Table S18. Clustering of the age-DE SAT ASPC genes by their expression profiles during a 14-day SAT ASPC differentiation (i.e. adipogenesis) experiment (see the excel file Additional File 3: Table S18.xlsx).

Table S19. Gene set enrichment analysis using EnrichR and the human subset of the ChIP Enrichment Analysis (CHEA) 2022 database identifies 5 subgroups significantly enriched for common transcription factor targets.

Subgrouping[A]	Transcription factor	Number of Overlapping Genes	Adjusted P-Value[B]
Group 1	ZNF217	4	2.01E-02
Group 1	FOXM1	3	2.01E-02
Group 2	NR3C1	10	6.59E-03
Group 2	MYC	7	2.57E-02
Group 2	ZNF217	6	2.57E-02
Group 2	PHOX2B	7	2.57E-02
Group 2	SOX2	8	2.57E-02
Group 2	TCF3/E2A	6	3.60E-02
Group 3	NFKB1	10	2.34E-03
Group 3	CTBP2	5	9.10E-03
Group 3	KDM2B	6	9.10E-03
Group 3	TCF4	8	9.10E-03
Group 3	PHOX2B	6	1.83E-02
Group 3	PPARD	7	1.95E-02
Group 3	ISL1	4	2.87E-02
Group 3	SMAD4	6	3.15E-02
Group 3	AR	5	3.16E-02
Group 3	TCF3/E2A	5	3.16E-02
Group 3	CTBP1	4	3.20E-02
Group 4	UBF1/2	5	4.64E-02
Group 4	SOX2	6	4.64E-02
Group 5	TCF4	7	1.86E-02
Group 5	GBX2	3	4.54E-02
Group 5	P300	6	4.62E-02
Group 5	TCF21	6	4.62E-02

[A] As determined using the DPGP tool to cluster the longitudinal bulk expression data from the ASPC differentiation experiment (See Fig. 4; Additional File 3: Table S18, S19).

[B] Benjamini-Hochberg adjusted p-value (FDR<0.05).

Table S20. Intercellular interactions involving the 76 age-DE SAT ASPC genes are significantly stronger ($p<0.05$) in the older individuals of the adipose snRNA-seq cohort compared to the younger individuals.

Cell-type[A]	Mean interaction score in older group	Mean interaction score in younger group	p-value[B]
ASPC	0.641	0.518	4.60E-46
Adipocyte	0.602	0.442	2.25E-07
B	0.551	0.509	5.21E-03
Endothelial	0.660	0.524	5.77E-07
Fibroblast	0.707	0.624	1.88E-06
LymphEndo	0.642	0.163	4.13E-04
Mast	0.583	0.496	1.59E-04
Myeloid	0.603	0.522	1.79E-05
NKT	0.618	0.465	2.26E-06
Perivascular	0.683	0.597	9.35E-09
T	0.624	0.495	1.08E-10

[A] Cell-type interacting with ASPCs.

[B] As assessed by a one-sided, paired Wilcoxon test.

Table S21. Interactions involving the gene *RORA* show the largest differences in strength between the younger and older individuals (see the excel file Additional File 3: Table S21.xlsx).

Table S22. Variance in body mass index (BMI) explained by the genome-wide and regional polygenic risk scores (PRSs).

Group	N	$R^{2\,A}$ of genome-wide PRS	R^2 of age-DE[B] regional PRS	R^2 of non-age-DE[C] regional PRS
All individuals	193,602	0.1059	4.55×10^{-3}	5.48×10^{-3}
Females	104,614	0.0793	3.66×10^{-3}	4.24×10^{-3}
Males	88,988	0.0795	3.83×10^{-3}	4.88×10^{-3}

[A] Variance in BMI explained by the BMI PRS.

[B] The age-DE regional PRS was built using all variants in the *cis*-regions (+/-500kb) of the autosomal age-DE ASPC genes differentially expressed (DE) by age.

[C] The non-age-DE regional PRS was built using all variants in the *cis*-regions (+/-500kb) of the autosomal ASPC marker genes not DE by age.

Table S23. Estimated effect of the interaction between the age-DE regional BMI PRS and age on BMI in the individuals with normal BMI (BMI<25) and in the obese individuals (BMI>=30) in the UK Biobank.

Group	N	BMI status	Coefficient[A]	p-value[B]
All	45,203	Normal	-4.39×10^{-4}	0.829
All	45,203	Obese	-5.29×10^{-3}	9.50×10^{-3}
Females	24,025	Normal	1.26×10^{-3}	0.627
Females	24,025	Obese	-6.97×10^{-3}	7.68×10^{-3}
Males	21,178	Normal	2.25×10^{-3}	0.413
Males	21,178	Obese	-5.94×10^{-5}	0.983

[A] Unstandardized β (regional PRS*age) in the linear model adjusted BMI ~ age + regional PRS + regional PRS*age.

[B] p-value of coefficient as assessed by a Wald test.

Table S24. Local variants within the age-DE SAT ASPC genes landing in ASPC open chromatin regions interact with age on BMI in obese individuals of the UK Biobank (n=45,203).

SNP	Interaction coefficient[A]	p-value[B]	Nearest gene[C]	Adjusted p-value[D]
rs17554931	-6.74E-03	3.42E-04	CFD	0.042
rs11631777	-2.73E-03	1.04E-02	RORA	1
rs10980494	2.07E-03	2.08E-02	SVEP1	1
rs775250	2.11E-03	2.37E-02	STAT6	1
rs782930	1.94E-03	3.02E-02	RORA	1
rs113282909	-3.52E-03	4.53E-02	APOD	1
rs17834271	-2.88E-03	4.80E-02	SERPINF1	1

[A] Unstandardized β(SNP*age) in the linear model adjusted BMI ~ age + SNP + SNP*age

[B] As assessed by a Wald-test.

[C] Nearest gene among the age-DE ASPC genes.

[D] Bonferroni corrected p-value.
